# A DAG Scheduling Scheme on Heterogeneous Computing Systems Using Tuple-Based Chemical Reaction Optimization

**DOI:** 10.1155/2014/404375

**Published:** 2014-06-24

**Authors:** Yuyi Jiang, Zhiqing Shao, Yi Guo

**Affiliations:** College of Information Science and Engineering, East China University of Science and Technology, Shanghai 200237, China

## Abstract

A complex computing problem can be solved efficiently on a system with multiple computing nodes by dividing its implementation code into several parallel processing modules or tasks that can be formulated as directed acyclic graph (DAG) problems. The DAG jobs may be mapped to and scheduled on the computing nodes to minimize the total execution time. Searching an optimal DAG scheduling solution is considered to be NP-complete. This paper proposed a tuple molecular structure-based chemical reaction optimization (TMSCRO) method for DAG scheduling on heterogeneous computing systems, based on a very recently proposed metaheuristic method, chemical reaction optimization (CRO). Comparing with other CRO-based algorithms for DAG scheduling, the design of tuple reaction molecular structure and four elementary reaction operators of TMSCRO is more reasonable. TMSCRO also applies the concept of constrained critical paths (CCPs), constrained-critical-path directed acyclic graph (CCPDAG) and super molecule for accelerating convergence. In this paper, we have also conducted simulation experiments to verify the effectiveness and efficiency of TMSCRO upon a large set of randomly generated graphs and the graphs for real world problems.

## 1. Introduction

Modern computer systems with multiple processors working in parallel may enhance the processing capacity for an application. The effective scheduling of parallel modules of the application may fully exploit the parallelism. The application modules may communicate and synchronize several times during the processing. The limitation of the overall application performance may be incurred by a large communication cost on heterogeneous systems with a combination of GPUs, multicore processors and CELL processors, or distributed memory systems. And an effective scheduling may greatly improve the performance of the application.

Scheduling generally defines not only the processing order of application modules but also the processor assignment of these modules. The concept of makespan (i.e., the schedule length) is used to evaluate the scheduling solution quality including the entire execution and communication cost of all the modules. On the heterogeneous systems [1–4], searching optimal schedules minimizing the makespan is considered as a NP-complete problem. Therefore, two classes of scheduling strategies have been proposed to solve this problem by finding the suboptimal solution with lower time overhead, such as heuristic scheduling and metaheuristic scheduling.

Heuristic scheduling strategies try to identify a good solution by exploiting the heuristics. An important subclass of heuristic scheduling is list scheduling with an ordered task list for a DAG job on the basis of some greedy heuristics. Moreover, the ordered tasks are selected to be allocated to the processors which minimize the start times in list scheduling algorithms. In heuristic scheduling, the attempted solutions are narrowed down by greedy heuristics to a very small portion of the entire solution space. And this limitation of the solution searching leads to the low time complexity. However, the higher complexity DAG scheduling problems have, the harder greedy heuristics produce consistent results on a wide range of problems, because the quality of the found solutions relies on the effectiveness of the heuristics, heavily.

Metaheuristic scheduling strategies such as ant colony optimization (ACO), genetic algorithms (GA), Tabu search (TS), simulated annealing (SA), and so forth take more time cost than heuristic scheduling strategies, but they can produce consistent results with high quality on the problems with a wide range by directed searching solution spaces.

Chemical reaction optimization (CRO) is a new metaheuristic method proposed very recently and has shown its power to deal with NP-complete problem. There is only one CRO-based algorithm called double molecular structure-based CRO (DMSCRO) for DAG scheduling on heterogeneous system as far as we know. DMSCRO has a better performance on makespan and convergence rate than genetic algorithm (GA) for DAG scheduling on heterogeneous systems. However, the rate of convergence of DMSCRO as a metaheuristic method is still defective. This paper proposes a new CRO-based algorithm, tuple molecular structure-based CRO (TMSCRO), for the mentioned problem, encoding the two basic components of DAG scheduling, module execution order and module-to-processor mapping, into an array of tuples. Combining this kind of molecular structure with the elementary reaction operator designed in TMSCRO has a better capability of intensification and diversification than DMSCRO. Moreover, in TMSCRO, the concept of constrained critical paths (CCPs) [5] and constrained-critical-path directed acyclic graph (CCPDAG) are applied to creating initial population in order to speed up the convergence of TMSCRO. In addition, the first initial molecule, InitS, is also considered to be a super molecule [6] for accelerating convergence, which is converted from the scheduling result of the algorithm constrained earliest finish time (CEFT).

In theory, a metaheuristic method will gradually approach the optimal result if it runs for long enough, based on No-Free-Lunch Theorem, which means the performances of the search for optimal solution of each metaheuristic algorithm are alike when averaged over all possible fitness functions. We have conducted the simulation experiments over the graphs abstracted from two well-known real applications: Gaussian elimination and molecular dynamics application and also a large set of randomly generated graphs. The experiment results show that the proposed TMSCRO can achieve similar performance as DMSCRO in the literature in terms of makespan and outperforms the heuristic algorithms.

There are three major contributions of this work.

1. Developing TMSCRO based on CRO framework by designing a more reasonable molecule encoding method and elementary chemical reaction operators on intensification and diversification search than DMSCRO.
2. For accelerating convergence, applying CEFT and CCPDAG to the data pretreatment, utilizing the concept of CCPs in the initialization, and using the first initial molecule, InitS, to be a super molecule in TMSCRO.
3. Verifying the effectiveness and efficiency of the proposed TMSCRO by simulation experiments. The simulation results of this paper show that TMSCRO is able to approach similar makespan as DMSCRO, but it finds good solutions faster than DMSCRO by 12.89% on average (by 26.29% in the best case).

## 2. Related Work

Most of the scheduling algorithms can be categorized into heuristic scheduling (including list scheduling, duplication-based scheduling, and cluster scheduling) and metaheuristic (i.e., guided-random-search-based) scheduling. These strategies are to generate the scheduling solution before the execution of the application. The approaches adopted by these different scheduling strategies are summarized in this section.

### 2.1. Heuristic Scheduling

Heuristic methods usually provide near-optimal solutions for a task scheduling problem in less than polynomial time. The approaches adopted by heuristic method search only one path in the solution space, ignoring other possible ones [7]. Three typical kinds of algorithms based on heuristic scheduling for the DAG scheduling problem are discussed as below, such as list scheduling [7, 8], cluster scheduling [9, 10], and duplication-based scheduling [11, 12].

The list scheduling [7, 13–21] generates a schedule solution in two primary phases. In phase 1, all the tasks are processed in a sequence order by their assigned priorities, which are normally based on the task execution and communication costs. There are two attributes used in most list scheduling algorithms, such as* b*-level and* t*-level, to assign task priorities. In a DAG,* b*-level of a node (task) is the length of the longest path from the end node to the node; however,* t*-level of a node is the length of the longest path from the entry node to the node. In phase 2, the processors are assigned to each task in the sequence.

The heterogeneous earliest finish time (HEFT) scheduling algorithm [16] assigns the scheduling task priorities based on the earliest start time of each task. HEFT allocates a task to the processor which minimizes the task's start time.

The modified critical path (MCP) scheduling [22] considers only one CP (critical path) of the DAG and assigns the scheduling priority to tasks based on their latest start time. The latest start times of the CP tasks are equal to their* t*-levels. MCP allocates a task to the processor which minimizes the task's start time.

Dynamic-level scheduling (DLS) [23] uses the concept of the dynamic level, which is the difference between the* b*-level and earliest start time of a task on a processor. Each time the (task, processor) pair with the largest dynamic-level value is chosen by DLS during the task scheduling.

Mapping heuristic (MH) [24] assigns the task scheduling priorities based on the static* b*-level of each task, which is the* b*-level without the communication costs between tasks. Then, a task is allocated to the processor which gives the earliest start time.

Levelized-min time (LMT) [17] assigns the task scheduling priority in two steps. Firstly, it groups the tasks into different levels based on the topology of the DAG, and then in each level, the task with the highest priority is the one with the largest execution cost. A task is allocated to the processor which minimizes the sum of the total communication costs with the tasks in the previous level and the task's execution cost.

There are two heuristic algorithms for DAG scheduling on heterogeneous systems proposed in [8]. One algorithm named HEFT_T uses the sum of* t*-level and* b*-level to assign the priority to each task. In HEFT_T, the critical tasks are attempted to be on the same processor, and the other tasks are allocated to the processor that gives earliest start time. The other algorithm named HEFT_B applies the concept of* b*-level to assign the priority (i.e., scheduling order) to each task. After the priority assignment, a task is allocated to the processor that minimizes the start time. The extensive experiment results in [8] demonstrate that HEFT_B and HEFT_T outperform (in terms of makespan) other representative heuristic algorithms in heterogeneous systems, such as DLS, MH, and LMT.

Comparing with the list scheduling algorithms, the duplication-based algorithms [23, 25–29] attempt to duplicate the tasks to the same processor on heterogeneous systems, because the duplication may eliminate the communication cost of these tasks and it may effectively reduce the total schedule length.

The clustering algorithms [8, 11, 30–32] regard task collections as clusters to be mapped to appropriate processors. These algorithms are mostly used in the homogeneous systems with unbounded number of processors and they will use as many processors as possible to reduce the schedule length. Then, if the number of the processors used for scheduling is more than that of the available processors, the task collections (clusters) are processed further to fit in with a limited number of processors.

### 2.2. Metaheuristic Scheduling

In comparison with the algorithms based on heuristic scheduling, the metaheuristic (guided-random-search-based) algorithms use a combinatorial process for solution searching. In general, with robust performance on many kinds of scheduling problems, the metaheuristic algorithms need sampling candidate solutions in the search space, sufficiently. Many metaheuristic algorithms have been applied to solve the task scheduling problem successfully, such as GA, chemical reaction optimization (CRO), energy-efficient stochastic [33], and so forth.

GA [15, 31, 34–36] is the mostly used metaheuristic method for DAG scheduling. In [15], a solution for scheduling is encoded as one-dimensional string representing an ordered list of tasks to be allocated to a processor. In each string of two parent solutions, the crossover operator selects a crossover point randomly and then merges the head portion of one parent with the tail portion of the other. Mutation operator exchanges two tasks in two solutions, randomly. The concept of makespan is used to evaluate the scheduling solution quality by fitness function.

Chemical reaction optimization (CRO) was proposed very recently [20, 30, 37–39]. It mimics the interactions of molecules in chemical reactions. CRO has good performance already in solving many problems, such as quadratic assignment problem (QAP), resource-constrained project scheduling problem (RCPSP), channel assignment problem (CAP) [39], task scheduling in grid computing (TSGC) [40], and 0-1 knapsack problem (KP01) [41]. So far as we know, double molecular structure-based chemical reaction optimization (DMSCRO) recently proposed in [37] is the only one CRO-based algorithm with two molecular structures for DAG scheduling on heterogeneous systems. CRO-based algorithm (just DMSCRO) mimics the chemical reaction process in a closed container and accords with energy conservation. In DMSCRO, one solution for DAG scheduling including two essential components, task execution order and task-to-processor mapping, corresponds to a double-structured molecule with two kinds of energy, potential energy (PE) and kinetic energy (KE). The value of PE of a molecule is just the fitness value (objective value), makespan, of the corresponding solution, which can be calculated by the fitness function designed in DMSCRO, and KE with a nonnegative value is to help the molecule escape from local optimums. There are four kinds of elementary reactions used to do the intensification and diversification search in the solution space to find the solution with the minimal makespan, and the principle of the reaction selection is in detail presented in Section 3.2. Moreover, a central buffer is also applied in DMSCRO for energy interchange and conservation during the searching progress. However, as a metaheuristic method for DAG scheduling, DMSCRO still has very large time expenditure and the rate of convergence of this algorithm needs to be improved. Comparing with GA, DMSCRO is similar in model and workload to TMSCRO proposed in this paper.

Our work is concerned with the DAG scheduling problems and the flaw of CRO-based method for DAG scheduling, proposing a tuple molecular structure-based chemical reaction optimization (TMSCRO). Comparing with DMSCRO, TMSCRO applies CEFT [5] to data pretreatment to take the advantage of CCPs as heuristic information for accelerating convergence. Moreover, the molecule structure and elementary reaction operators design in TMSCRO are more reasonable than those in DMSCRO on intensification and diversification of searching the solution space.

## 3. Background

### 3.1. CEFT

Constrained earliest finish time (CEFT) based on the constrained critical paths (CCPs) was proposed for heterogeneous system scheduling in [5]. In contrast to other approaches, the CEFT strategy takes account of a broader view of the input DAG. Moreover, the CCPs can be scheduled efficiently because of their static generation.

The constrained critical path (CCP) is a collection with the tasks ready for scheduling only. A task is ready when all its predecessors were processed. In CEFT, a critical path (CP) is generally the longest path from the start node to the end node for scheduling in the DAG. The DAG is initially traversed and critical paths are found. Then it is pruned off the nodes that constitute a critical path. The subsequent traversals of the pruned graph produce the remaining critical paths. While the nodes are being removed from the task graph, a pseudo-edge to the start or end node is added if a node has no predecessors or no successors, respectively. The CCPs are subsequently formed by selecting ready nodes in the critical paths in a round-robin fashion. Each CCP may be assigned a single processor which has the minimum finish time of processing all the tasks in the CCP. All the tasks in a CCP not only reduce the communication cost, but also benefit from a broader view of the task graph.

Consider the CEFT algorithm generates schedules for n tasks with |*P*| heterogeneous processors. Some specific terms and their usage are indicated in Table 1.

The CEFT scheduling approach (Algorithm 1) works in two phases. (1) The critical paths are generated according to the description in the second paragraph of Section  3.1. The critical paths are traversed and the ready nodes are inserted into the constrained critical paths (CCPs) CCP_*j*_, ∀*j* = 1,2,…, |*Q*|. If no more ready nodes are in a critical path, the constrained critical path takes nodes from the next critical path following round-robin traversal of the critical paths. (2) All the CCPs are traversed in order (line 12). Then, ST_*P*_*r*__(*w*, *k*), the maximum of AT_*P*_*r*__ and the start time of the predecessors of each node *w*, is calculated (1). EFT_*P*_*r*__(*w*) is computed as the sum of ST_*P*_*r*__(*w*, *k*) and EC_*P*_*r*__(*w*) (2). *E*_*P*_*r*__(*Q*_*j*_) is the maximum of the finish times of all the CCP nodes on the same processor *P*_*r*_ (3). The processor is then assigned to constrained-critical-path CCP_*j*_ which minimizes the CEFT_*P*_*r*__(CCP_*j*_) value (line 20). After the actual finish time AEFT_*w*_ of each task *w* in CCP_*j*_ is updated, the processor assignment continues iteratively.

### 3.2. CRO

Chemical reaction optimization (CRO) mimics the process of a chemical reaction where molecules undergo a series of reactions between each other or with the environment in a closed container. The molecules are manipulated agents with a profile of three necessary properties of the molecule, including the following. (1) The molecular structure *S*: *S* actually structure represents the positions of atoms in a molecule. Molecular structure can be in the form of a number, a vector, a matrix, or even a graph which is independent of the problem, (2) (Current) potential energy (PE):  PE is the objective function value of the current molecular structure *ω*, that is, PE_*ω*_ = *f*(*ω*). (3) (Current) kinetic energy (KE): KE is a nonnegative number and it helps the molecule escape from local optimums. There is a central energy buffer implemented in CRO. The energy in CRO may accord with energy conservation and can be exchanged between molecules and the buffer.

Four kinds of elementary reactions may happen in CRO, which are defined as below.

(1) On-wall ineffective collision: on-wall ineffective collision is a unimolecule reaction with only one molecule. In this reaction, a molecule *ω* is allowed to change to another one *ω*′, if their energy values accord with the following inequality:
  (1) $$\begin{array}{c} {\text{PE}}_{\omega }+{\text{KE}}_{\omega }\geq {\text{PE}}_{\omega^{\prime }}; \end{array}$$
  after this reaction, KE will be redistributed in CRO. The redundant energy with the value KE_*ω*′_ = (PE_*ω*_ + KE_*ω*_ − PE_*ω*′_) × *t* will be stored in the central energy buffer. Parameter t is a random number from KELossRate to 1 and KELossRate, a system parameter set during the CRO initialization, is the KE loss rate less than 1.
(2) Decomposition: decomposition is the other unimolecule reaction in CRO. A molecule *ω* may decompose into two new molecules, *ω*_1_′ and *ω*_2_′, if their energy values accord with inequality (2), in which buf denotes the energy in the buffer, representing the energy interactions between molecules and the central energy buffer:
  (2) $$\begin{array}{c} {\text{PE}}_{\omega }+{\text{KE}}_{\omega }+\text{buf}\geq {\text{PE}}_{\omega_{1}^{\prime }}+{\text{PE}}_{\omega_{2}^{\prime }}; \end{array}$$
  after this reaction, buf is updated by (3) and the KEs of *ω*_1_′ and *ω*_2_′ are, respectively, computed as (4) and (5), where Edecomp = (PE_*ω*_ + KE_*ω*_)−(PE_*ω*_1_′_ + PE_*ω*_2_′_) and  *μ*1, *μ*2, *μ*3, *μ*4 is a number randomly selected from the range of [0, 1]. Consider
  (3) buf=Edecomp+buf−(PEω1′+PEω2′),
  (4) KEω1′=(Edecomp+buf)×μ1×μ2,
  (5) KEω2′=(Edecomp+buf−KEω1′)×μ3×μ4.
(3) Intermolecular ineffective collision: intermolecular ineffective collision is an intermolecule reaction with two molecules. Two molecules, *ω*_1_ and *ω*_2_, may change to two new molecules, *ω*_1_′ and *ω*_2_′, if their energy values accord with the following inequality:
  (6) $$\begin{array}{c} {\text{PE}}_{\omega_{1}}+{\text{PE}}_{\omega_{2}}+{\text{KE}}_{\omega_{1}}+{\text{KE}}_{\omega_{2}}\geq {\text{PE}}_{\omega_{1}^{\prime }}+{\text{PE}}_{\omega_{2}^{\prime }}; \end{array}$$
  after this reaction, the KEs of *ω*_1_′ and *ω*_2_′, KE_*ω*_1_′_ and KE_*ω*_2_′_, will share the spare energy Eintermole calculated by (7). KE_*ω*_1_′_ and KE_*ω*_2_′_ are computed as (8) and (9), respectively, where  *μ*1 is a number randomly selected from the range of [0, 1]. Consider
  (7) Eintermole=(PEω1+PEω2+KEω1+KEω2)−(PEω1′+PEω2′),
  (8) $$\begin{array}{c} {\text{KE}}_{\omega_{1}^{\prime }}=\text{Eintermole}\times \mu 1, \end{array}$$
  (9) $$\begin{array}{c} {\text{KE}}_{\omega_{2}^{\prime }}=\text{Eintermoler}\times \left(1-\mu 1\right). \end{array}$$
(4) Synthesis: synthesis is also an intermolecule reaction. Two molecules, *ω*_1_ and *ω*_2_, may be combined to a new molecule, *ω*′, if their energy values accord with inequality (10). The KE of *ω*′ is computed as (11):
  (10) $$\begin{array}{c} {\text{PE}}_{\omega_{1}}+{\text{PE}}_{\omega_{2}}+{\text{KE}}_{\omega_{1}}+{\text{KE}}_{\omega_{2}}\geq {\text{PE}}_{\omega^{\prime }}, \end{array}$$
  (11) $$\begin{array}{c} {\text{KE}}_{\omega^{\prime }}={\text{PE}}_{\omega_{1}}+{\text{PE}}_{\omega_{2}}+{\text{KE}}_{\omega_{1}}+{\text{KE}}_{\omega_{2}}-{\text{PE}}_{\omega^{\prime }}. \end{array}$$

The canonical CRO works as follows. Firstly, the initialization of CRO is to set system parameters, such as PopSize (the size of the molecules), KELossRate, InitialKE (the initial energy of molecules), buf (initial energy in the buffer), and MoleColl (MoleColl is a threshold value to determine whether to perform a unimolecule reaction or an intermolecule reaction). Then the CRO processes a loop. In each iteration, whether to perform a unimolecule reaction or an intermolecule reaction is first decided in the following way. A number *ε* is randomly selected from the range of [0, 1]. If *ε* is bigger than MoleColl, a unimolecule reaction will be chosen, or an intermolecular reaction is to occur. If it is a unimolecular reaction, a parameter *θ* as a threshold value is used to guide the further choice of on-wall collision or decomposition. NumHit is the parameter used to record the total collision number of a molecule. It will be updated after a molecule undergoes a collision. If the NumHit of a molecule is larger than *θ*, a decomposition will then be selected. Similarly, a parameter *ϑ* is used to further decide selection of an intermolecule collision reaction or a synthesis reaction. *ϑ* specifies the least KE of a molecule. Synthesis reaction will be chosen when both KEs of the molecules *ω*_1_ and *ω*_2_ are less than *ϑ*, or intermolecular ineffective collision reaction will take place. When the stopping criterion satisfies (e.g., a better solution cannot be found after a certain number of consecutive iterations), the loop will be stopped and the best solution is just the molecule that possesses the lowest PE.

## 4. Models

This section discusses the system, application, and task scheduling model assumed in this work. The definition of the notations can be found in the Notations section.

### 4.1. System Model

In this paper, there are multiple heterogeneous processors in the target system, which are presented by *P* = {*p*_*i*_∣*i* = 1,2, 3,…, |*P*|}. They are fully interconnected with high speed network. Each task in a DAG can only be executed on one processor on heterogeneous system. The edges of the graph are labeled with communication cost that should be taken into account if its start and end tasks are executed on different processors. The communication cost is zero when the same processor is assigned to two communicating modules.

We assume a static computing system model in which the constrained relations and the execution costs of tasks are known a priori and the execution and communication can be performed simultaneously by the processors. In this paper, the heterogeneity is represented by EC_*P*_*r*__(*w*), which means the execution cost of a node w using processor *P*_*r*_. As the assumption of the MHM model, the heterogeneity in the simulations is set as follows to make a processor have different speed for different tasks. The value of each EC_*P*_*r*__(*w*) is randomly chosen within the scope of [1 − *g*%, 1 + *g*%] by using a parameter *g*  (*g* ∈ (0,1)). Therefore, the heterogeneity level can be formulated as (1 + *g*%)/(1 − *g*%). *g* is set as the value that makes the heterogeneity level 2 in this paper unless otherwise specified.

### 4.2. Application Model

In DAG scheduling, finding optimal schedules is to find the scheduling solution with the minimum schedule length. The schedule length encompasses the entire execution and communication cost of all the modules and is also termed as makespan. In this paper, the task scheduling problem is to map a set of tasks to a set of processors, aiming at minimizing the makespan. It takes as input a directed acyclic graph DAG = (*V*, *E*), with |*V*| nodes representing tasks, and |*E*| edges representing constrained relations among the tasks. *V* = (*v*_1_, *v*_2_,…, *v*_*i*_,…, *v*_|*V*|_) is a node sequence in which the hypothetical entry node (with no predecessors) *v*_1_ and end node (with no successors) *v*_|*V*|_, respectively, represent the beginning and the end of execution. The execution cost value of *v*_*i*_ on processor *p*_*k*_ is denoted as EC_*p*_*k*__(*v*_*i*_), and the average computation cost of *v*_*i*_, denoted as $\overset{-}{W(v_{i})}$, can be calculated by (12). The parameter for the amounts of computing power available at each node in a heterogeneous system and its heterogeneous level value is given in the 5th paragraph of Section 6 and Table 1.

**Figure 1:**
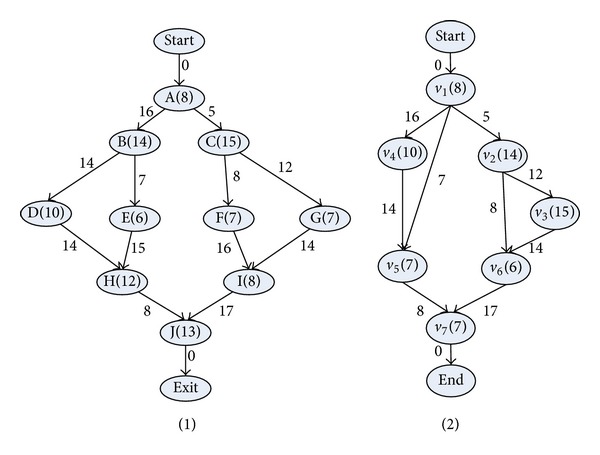
Two simple DAG models with 7 and 10 tasks.

**Figure 2:**
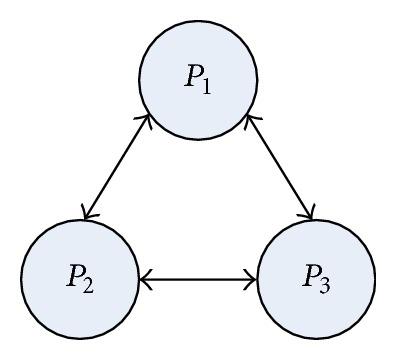
A fully connected parallel system with 3 heterogeneous processors.

*E* = {*E*_*i*_∣*i* = 1,2, 3,…, |*E*|} is an edge set in which *E*_*i*_ = (ev_*s*_, ev_*e*_, ew_*s*,*e*_), with ev_*s*_  &  ev_*e*_ ∈ {*v*_1_, *v*_2_,…, *v*_|*V*|_} representing its start and end nodes, and the value of communication cost between ev_*s*_  and ev_*e*_ is denoted as ew_*s*,*e*_. The DAG topology of an exemplar application model and system model is shown in Figures 1 and 2, respectively.

Consider

(12) $$\begin{array}{c} \overset{-}{W\left(v_{i}\right)}=\overset{\left|P\right|}{\underset{k=1}{\sum }}\frac{{\text{EC}}_{p_{k}}\left(v_{i}\right)}{\left|P\right|}. \end{array}$$

The constrained-critical-path sequence of DAG = (*V*, *E*) is denoted as CCP = (CCP_1_, CCP_2_,…, CCP_|CCP|_) with CCP_*i*_ = (cv_*i*,1_, cv_*i*,2_,…, cv_*i*,|CCP_*i*_|_) in which the set {cv_*i*,1_, cv_*i*,2_,…, cv_*i*,|CCP_*i*_|_}⊆{*v*_1_, *v*_2_,…, *v*_|*V*|_}.

The start time of the task *v*_*i*_ on processor *p*_*k*_ is denoted as ST_*p*_*k*__(*v*_*i*_), which can be calculated using (13), where Pred(*v*_*i*_) is the set of the predecessors of the task *v*_*i*_. And the earliest finish time of the task *v*_*i*_ on processor *p*_*k*_ is denoted as EFT_*p*_*k*__(*v*_*i*_), which can be calculated using (14):

(13) $$\begin{array}{c} {\text{ST}}_{p_{k}}\left(v_{i}\right)=\{\begin{array}{cc} 0, & v_{i}=v_{1}\\ \underset{v_{j}\in \text{Pred}\left(v_{i}\right)}{\max}{\text{EFT}}_{p_{k}}\left(v_{i}\right), & p_{k}=p_{m}\\ \underset{v_{j}\in \text{Pred}\left(v_{i}\right)}{\max}\left({\text{EFT}}_{p_{m}}\left(v_{j}\right)+{\text{ew}}_{j,i}\right), & p_{k}\neq p_{m} \end{array} \end{array}$$

(14) $$\begin{array}{c} {\text{EFT}}_{p_{k}}\left(v_{i}\right)={\text{ST}}_{p_{k}}\left(v_{i}\right)+{\text{EC}}_{p_{k}}\left(v_{i}\right). \end{array}$$

The communication to computation ratio (CCR) can be used to indicate whether a DAG is communication intensive or computation intensive. For a given DAG, it is computed by the average communication cost divided by the average computation cost on a target computing system. The computation can be formulated as follows:

(15) $$\begin{array}{c} \text{CCR}=\frac{\sum_{(v_{i},v_{j},\text{e}{\text{w}}_{i,j})\in E}{\text{ew}}_{i,j}}{\overset{-}{W(v_{i})}}. \end{array}$$

## 5. Design of TMSCRO

TMSCRO mimics the interactions of molecules in chemical reactions with the concepts of molecule, atoms, molecular structure, and energy of a molecule. The structure of a molecule is unique, which represents the atom positions in a molecule. The interactions of molecules in four kinds of basic chemical reactions, on-wall ineffective collision, decomposition, intermolecular ineffective collision, and synthesis, aim to transform to the molecule with more stable states which has lower energy. In DAG scheduling, a scheduling solution including a task and processor allocation corresponds to a molecule in TMSCRO. This paper also designs the operators on the encoded scheduling solutions (tuple arrays). These designed operators correspond to the chemical reactions and change the molecular structures. The arrays with different tuples represent different scheduling solutions, and we can calculate the corresponding makespan of the scheduling solution. A scheduling solution makespan corresponds to the energy of a molecule.

In this section, we first present the data pretreatment of the TMSCRO. After the presentation of the encoding of scheduling solutions and the fitness function used in the TMSCRO, we present the design of four elementary chemical reaction operators in each part of the TMSCRO. Finally, we outline the framework of the TMSCRO scheme and discuss a few important properties in TMSCRO.

### 5.1. Molecular Structure, Data Pretreatment, and Fitness Function

This subsection first presents the encoding of scheduling solutions (i.e., the molecular structure) and data pretreatment, respectively. Then we give the statement of the fitness function for optimization designed in TMSCRO.

#### 5.1.1. Molecular Structure and Data Pretreatment

A reasonable initial population in CRO-based methods may increase the scope of searching over the fitness function [20] to support faster convergence and to result in a better solution. Constrained critical paths (CCPs) can be seen as the classification of task sequences constructed by constrained earliest finish time (CEFT) algorithm, which takes into account all factors in DAG (i.e., the average of each task execution cost, the communication costs, and the graph topology). Therefore, TMSCRO utilizes the CCPs to create a reasonable initial population based on a broad view of DAG.

The data pretreatment is to generate the CCPDAG from DAG and to construct CCPS for the initialization of TMSCRO. The CCPDAG is a directed acyclic graph with |CCP| nodes representing constrained critical paths (CCP_*s*_), two virtual nodes (i.e., start and end) representing the beginning and exit of execution, respectively, and |CE| edges representing dependencies among the nodes. The edges of CCPDAG are not labeled with communication overhead which is different from DAG. The data pretreatment includes two steps.

1. The CCP and the processor allocation of each element of CCP in DAG can be obtained by executing CEFT and the first initial CCP solution, InitCCPS = ((CCP_1_, sp_1_), (CCP_2_, sp_2_),…, (CCP_|CCP|_, sp_|CCP|_)), can also be got, in which ((CCP_*i*_, sp_*i*_)) is sorted as the generated order of CCP_*i*_ and sp_*i*_ is processor assignment of CCP_*i*_ after executing CEFT. Consider the graph as shown in Figure 1; the resulting CCPs are indicated in Table 2.
2. After the execution of CEFT for DAG, the CCPDAG is generated with the input of CCP and DAG. A detailed description is given in Algorithm 2.

**Figure 3:**
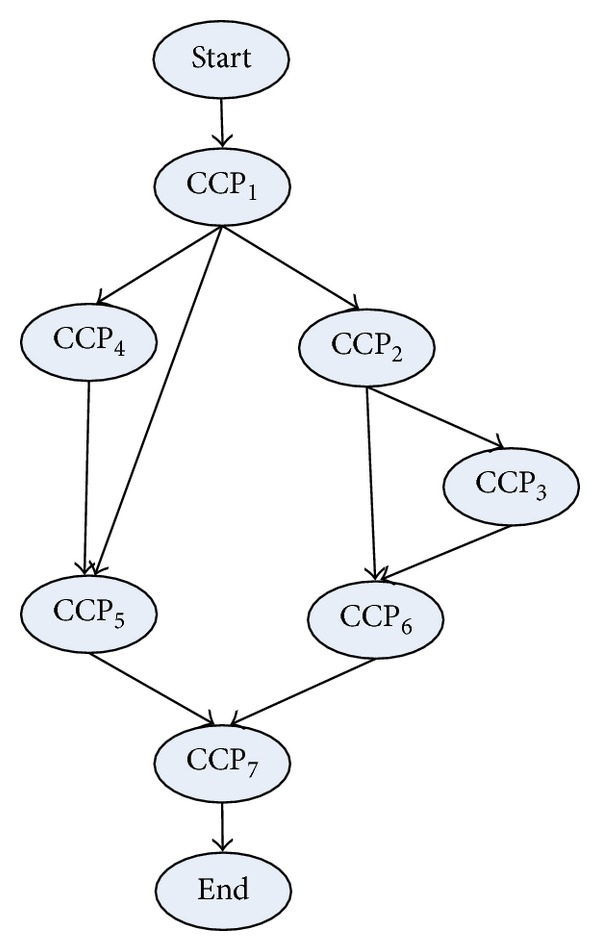
CCPDAG corresponding to the DAG as shown in Figure 1 and the CCP as indicated in Table 1.

As shown in Algorithm 1, the edge *E*_*i*_ of DAG with the start node CCP_*s*_ and the end node CCP_*e*_ is obtained in each loop (line 1). BelongCCP(*v*_*i*_) represents which CCP_*j*_ in CCP*v*_*i*_ belongs to (line 2 and line 3). If CCP_*s*_ and CCP_*e*_ are different CCPs and there is no edge between them (line 4), then the edge between CCP_*s*_ and CCP_*e*_ is generated (line 5). Finally, the nodes, start and end, and the edges among them and CCP nodes are added (line 7, line 8, and line 9). Consider the DAG as shown in Figure 1 and the CCP as indicated in Table 1. The resulting CCPDAG is shown in Figure 3.

In this paper, there are two kinds of molecular structures of TMSCRO, CCPS, and S. CCP molecular structure CCPS is just used in the initialization of TMSCRO, which can be formulated as in (16). Whereas the reaction molecular structure *S* converted from CCPS is used to participate in the elementary reaction of TMSCRO. In CCPS, ((CCP_*i*_, sp_*i*_))s are sorted as the topology of CCPDAG in which CCP_*i*_ is constrained critical path (CCP), and sp_*i*_ is the processor assigned to CCP_*i*_. |CCP | ≤ | *V*| because the number of elements in each SCCP_*i*_ is greater than or equal to one. A reaction molecule *S* can be formulated as in (17), which consists of an array of atoms (i.e., tuples) representing a solution of DAG scheduling problem. A tuple includes three integers *v*_*i*_, *f*_*i*_, and *p*_*i*_. The reaction molecular structure *S* is encoded with each integer in the permutation representing a task in DAG, the constraint relationship between a tuple and the one before it, and the processor *p*_*i*_. In each reaction molecular structure *S*, *v*_*i*_ represents a task in DAG and (*v*_1_, *v*_2_,…, *v*_|*V*|_) is a topological sequence of DAG. In *S*, if *v*_*A*_ of the tuple *A*, which is before tuple *B*, is the predecessor of *v*_*B*_ of tuple *B* in DAG, the second integer of tuple *B*, *f*_*B*_, will be 1, or it will be 0.  *p*_*i*_ represents the processor allocation of each *v*_*i*_ in the tuple. The sequence of the tuples in a reaction molecular structure *S* represents the scheduling order of each task in DAG:

(16) CCPS=((CCP1,sp1),(CCP2,sp2),…,(CCP|CCP|,sp|CCP|)),

(17) $$\begin{array}{c} S=\left(\left(v_{1},f_{1},p_{1}\right),\left(v_{2},f_{2},p_{2}\right),\ldots ,\left(v_{\left|V\right|},f_{\left|V\right|},p_{\left|V\right|}\right)\right). \end{array}$$

#### 5.1.2. Fitness Function

The initial molecule generator is used to generate the initial solutions for TMSCRO to manipulate. The first molecule InitS is converted from InitCCPS. Part three sp_*i*_ of each tuple is generated by a random perturbation in the first InitCCPS. A detailed description is given in Algorithms 3 and 4 and presents how to convert a CCPS to an S.

Potential energy (PE) is defined as the objective function (fitness function) value of the corresponding solution represented by S. The overall schedule length of the entire DAG, namely, makespan, is the largest finish time among all tasks, which is equivalent to the actual finish time of the end node in DAG. For the DAG scheduling problem by TMSCRO, the goal is to obtain the scheduling that minimizes makespan and ensure that the precedence of the tasks is not violated. Hence, each fitness function value is defined as

(18) $$\begin{array}{c} {\text{PE}}_{S}=\text{makespan}=\text{Fit}\left(S\right). \end{array}$$

Algorithm 5 presents how to calculate the value of the optimization fitness function Fit(*S*).

### 5.2. Elementary Chemical Reaction Operators

This subsection presents four elementary chemical reaction operators for sequence optimization and processor allocation optimization designed in TMSCRO, including on-wall collision, decomposition, intermolecular collision, and synthesis.

#### 5.2.1. On-Wall Ineffective Collision

**Figure 4:**
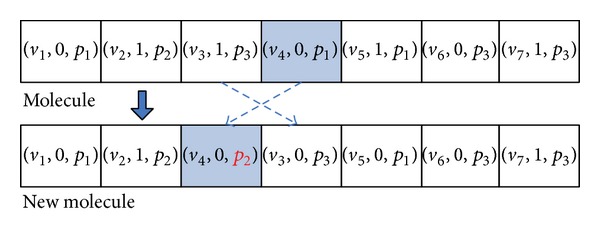
Illustration of molecular structure change for on-wall ineffective collision.

**Figure 5:**
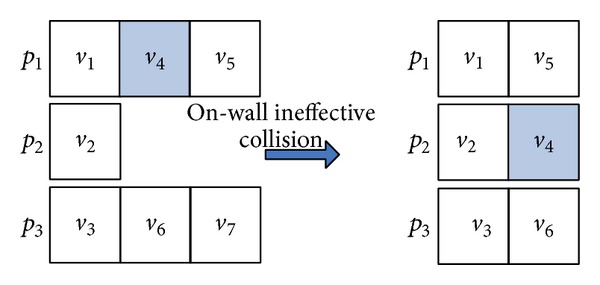
Illustration of the task-to-computing-node mapping for on-wall ineffective collision.

In this paper, the operator, OnWallT, is used to generate a new molecule *S*′ from a given reaction molecule *S* for optimization. OnWallT works as follows. (1) The operator randomly chooses a tuple (*v*_*i*_, *f*_*i*_, *p*_*i*_) with *f*_*i*_ = 0 in *S* and then exchanges the positions of (*v*_*i*_, *f*_*i*_, *p*_*i*_) and (*v*_*i*−1_, *f*_*i*−1_, *p*_*i*−1_). (2) *f*_*i*−1_,  *f*_*i*_ and *f*_*i*+1_ in *S* are modified as defined in the last paragraph of Section  5.1.1. (3) The operator changes *p*_*i*_ randomly. In the end, the operator generates a new molecule *S*′ from *S* as an intensification search. Figures 4 and 5 show the example which is the molecule corresponding to the DAG as shown in Figure 1(2).

#### 5.2.2. Decomposition

**Figure 6:**
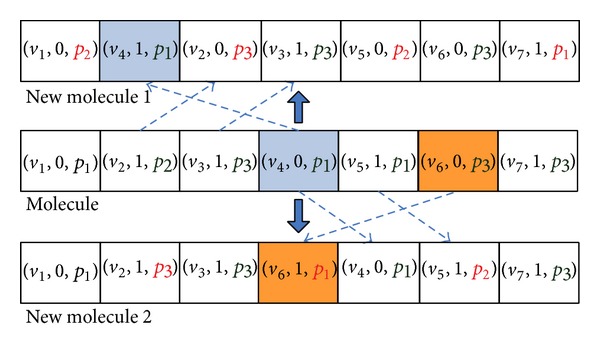
Illustration of molecular structure change for decomposition.

**Figure 7:**
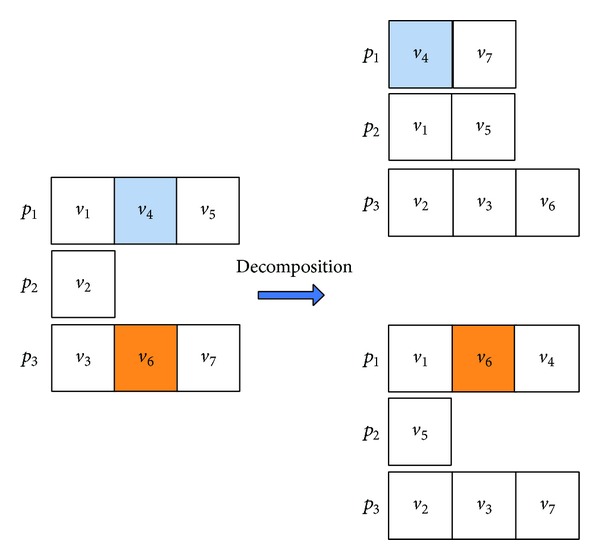
Illustration of the task-to-computing-node mapping for decomposition.

In this paper, the operator, DecompT, is used to generate new molecules S_1_′ and S_2_′ from a given reaction molecule *S*. DecompT works as follows. (1) The operator randomly chooses two tuples (tuples) (*v*_*i*_, *f*_*i*_, *p*_*i*_) with *f*_*i*_ = 0 and (*v*_*t*_, *f*_*t*_, *p*_*t*_) with *f*_*t*_ = 0 in *S* and then finds the tuple with the first predecessor of (*v*_*i*_, *f*_*i*_, *p*_*i*_), such as (*v*_*j*_, *f*_*j*_, *p*_*j*_), from the selection position to the beginning of reaction molecule *S*. (2) A random number *k* ∈ [*j* + 1, *i* − 1] is generated, and the tuple (*v*_*i*_, *f*_*i*_, *p*_*i*_) is stored in a temporary variable temp, and then from the position *i* − 1, the operator shifts each tuple by one place to the right position until a position *k*. (3) The operator moves the tuple temp to the position *k*. The rest of the tuples in *S*_1_′ are the same as those in *S*. (4) *f*_*i*_, *f*_*i*+1_ and *f*_*k*_ in *S* are modified as defined in the last paragraph of Section 5.1.1. (5) The operator generates the other new molecule *S*_2_′ as the former steps. The only difference is that, in step 2, we use (*v*_*t*_, *f*_*t*_, *p*_*t*_) instead of (*v*_*i*_, *f*_*i*_, *p*_*i*_). (6) The operator keeps the tuples in *S*_1_′, which is at the odd position in *S*, and retains the tuples in *S*_2_′, which is at the even position in *S*, and then changes the remaining *p*_*x*_ *s* of tuples in *S*_1_′' and *S*_2_′, randomly. In the end, the operator generates two new molecules *S*_1_′ and *S*_2_′ from *S* as a diversification search. Figures 6 and 7 show the example which is the molecule corresponding to the DAG as shown in Figure 1(2).

#### 5.2.3. Intermolecular Ineffective Collision

**Figure 8:**
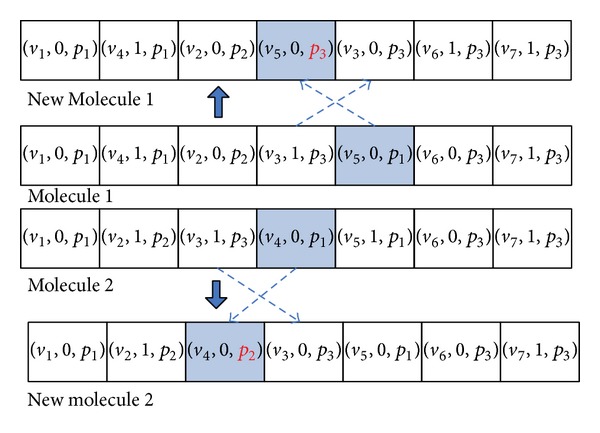
Illustration of molecular structure change for intermolecular ineffective collision.

**Figure 9:**
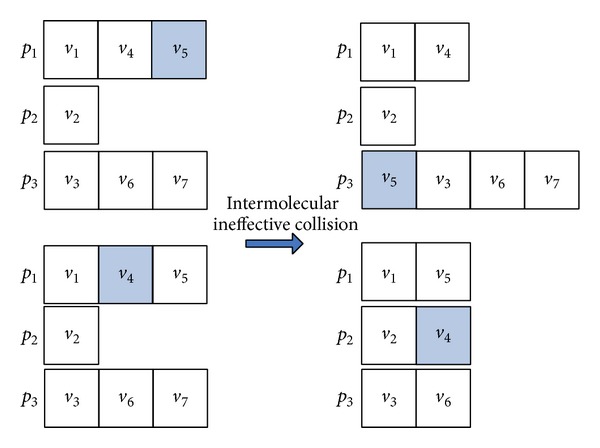
Illustration of the task-to-computing-node mapping for intermolecular ineffective collision.

In this paper, the operator, IntermoleT, is used to generate new molecules *S*_1_′ and *S*_2_′ from given molecules *S*_1_ and *S*_2_. This operator first uses the steps in OnWallT to generate *S*_1_′ from *S*_1_, and then the operator generates the other new molecule *S*_2_′ from *S*_2_ in similar fashion. In the end, the operator generates two new molecules *S*_1_′ and *S*_2_′ from *S*_1_ and *S*_2_ as an intensification search. Figures 8 and 9 show the example which is the molecule corresponding to the DAG as shown in Figure 1(2).

#### 5.2.4. Synthesis

**Figure 10:**
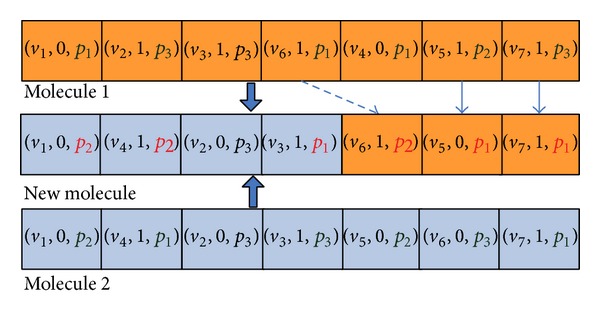
Illustration of molecular structure change for synthesis.

**Figure 11:**
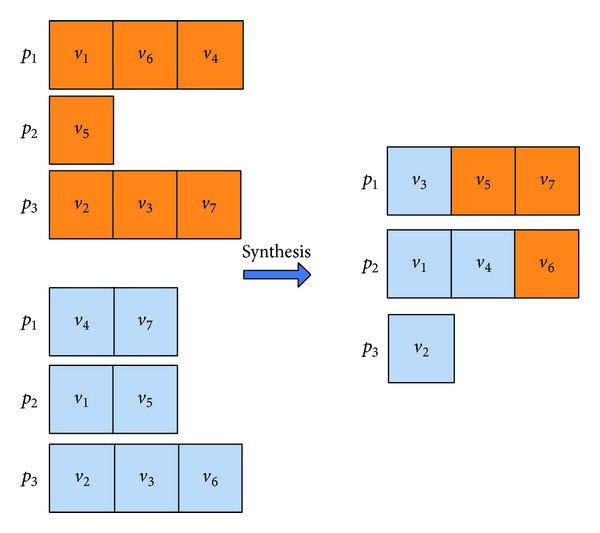
Illustration of the task-to-computing-node mapping for synthesis.

In this paper, the operator, SynthT, is used to generate a new molecule *S*′ from given molecules *S*_1_ and *S*_2_ for optimization. SynthT works as follows. (1) If |*V*| is plural, then the integer *i* = |*V* | /2; else *i* = (|*V*| + 1)/2. (2) *S*_1_ and *S*_2_ are cut off at the position *i* to become the left and right segments. (3) The left segments of *S*′ are inherited from the left segments of *S*_1_, randomly. (4) Each tuple in the right segments of *S*′ comes from the tuples in *S*_2_ that do not appear in the left segment of *S*′, with their *f*_*x*_ modified as defined in the last paragraph of Section  5.1.1 as well. (5) The operator keeps the tuples in *S*′, which are at the same position in *S*_1_ and *S*_2_ with the same *p*_*x*_ *s*, and then changes the remaining *p*_*y*_ *s* in *S*′, randomly. As a result, the operator generates *S*′ from *S*_1_ and *S*_2_ as a diversification search. Figures 10 and 11 show the example which is the molecule corresponding to the DAG as shown in Figure 1(2).

### 5.3. The Framework and Analysis of TMSCRO

The framework of TMSCRO is shown as an outline to schedule a DAG job in Algorithm 6 and the output of Algorithm 6 is just the resultant near-optimal solution for the corresponding DAG scheduling problem. In this framework, TMSCRO first initializes the process. Then, the process enters a loop. In each iteration, one of the elementary chemical reaction operators for optimization is performed to generate new molecules and PE of newly generated molecules will be calculated. The whole working of TMSCRO for DAG scheduling on heterogeneous problem is as presented in the last paragraph in Section  3.2. However, InitS is considered to be a super molecule [6], so it will be tracked and only participates in on-wall ineffective collision and intermolecular ineffective collision to explore as much as possible the solution space in its neighborhoods and the main purpose is to prevent InitS from changing dramatically. The iteration repeats until the stopping criteria are met. The stopping criteria may be set based on different parameters, such as the maximum amount of CPU time used, the maximum number of iterations performed, an objective function value less than a predefined threshold obtained, and the maximum number of iterations performed without further performance improvement. The stopping criterion of TMSCRO in the experiments of this paper is that the makespan is not changed after 5000 consecutive iterations in each loop. The time complexity of TMSCRO is *O*(iters × [2 × (|*V*|^2^ + |*E* | ×|*P*|)], where iters is the number of iterations in TMSCRO, respectively.

It is very difficult to theoretically prove the optimality of the CRO (as well as DMSCRO and TMSCRO) scheme [37]. However, by analyzing the molecular structure, chemical reaction operators, and the operational environment in TMSCRO, it can be shown to some extent that TMSCRO scheme has the advantage of three points in comparison with GA, SA, and DMSCRO.

First, just like DMSCRO, TMSCRO enjoys the advantages of GA and SA to some extent by analyzing the chemical reaction operators designed in TMSCRO and the operator environment of TMSCRO: (1) the OnWallT and IntermoleT in TMSCRO exchange the partial structure of two different molecules like the crossover operator in GA. (2) The energy conservation requirement in TMSCRO is able to guide the searching of the optimal solution in a similar way as the Metropolis Algorithm of SA guides the evolution of the solutions in SA. Second, constrained earliest finish time (CEFT) algorithm constructs constrained critical paths (CCPs) by taking into account a broader view of the input DAG [5]. TMSCRO applies CEFT and CCPDAG to the data pretreatment and utilizes CCPs in the initialization of TMSCRO to create a more reasonable initial population than DMSCRO for accelerating convergence, because a wide distributed initial population in CRO-based methods may increase the scope of searching over the fitness function [20] to support faster convergence and to result in a better solution. Moreover, to some degree, InitS is also similar to the super molecule in super molecule-based CRO or the “elite” in GA [6]. However, the “elite” in GA is usually generated from two chromosomes, while InitS is based on the whole input DAG by executing CEFT. Third, the operators with the molecular structure in TMSCRO are designed more reasonably than DMSCRO. In CRO-based algorithm, the operators of on-wall collision and intermolecular collision are used for intensifications, while the operators of decomposition and synthesis are for diversifications. The better the operator can get the better the search results of intensification and diversification are. This feature of CRO is very important, which gives CRO more opportunities to jump out of the local optimum and explore the wider areas in the solution space. In TMSCRO, the operators of OnWallT and IntermoleT every time only exchange the positions of one tuple and its former neighbor in the molecule with better capability of intensification on sequence optimization than DMSCRO, of which the reaction operators, OnWall (*ω*_1_) and Intermole (*ω*_1_, *ω*_2_) [37] (*ω*_1_ and *ω*_2_ are big molecules in DMSCRO), may change the task sequence(s) dramatically. Moreover, under the consideration that the optimization includes not only sequence but also processor assignment optimization, all reaction operators in TMSCRO can change the processor assignment, but DMSCRO has only two reactions, on-wall and synthesis [37], for processor assignment optimization. On the one hand, TMSCRO has 100% probability of searching the processor assignment solution space by four elementary reactions, with better capability of diversification and intensification on processor assignment optimization than DMSCRO, of which the chance to search this kind of solution space is only 50%. On the other hand, the division of diversification and intensification of four reactions in TMSCRO is very clear; however, this is not in DMSCRO. In each iteration, the diversification and intensification search in TMSCRO have the same probability to be conducted, whereas the possibility of diversification or intensification search in DMSCRO is uncertainty. This design enhances the ability to get better rapidity of convergence and search result in the whole solution space, which is demonstrated by the experimental results in Section 6.3.

## 6. Simulation and Results

**Figure 12:**
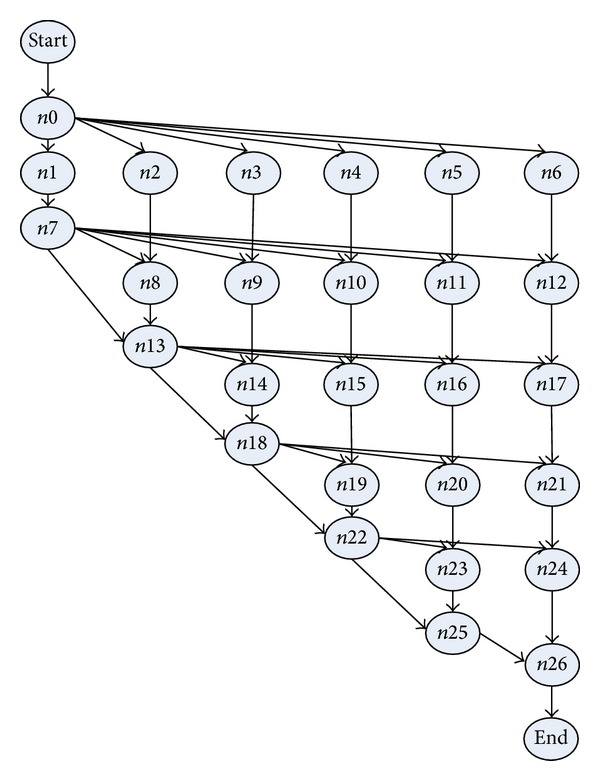
Gaussian elimination for a matrix of size 7.

**Figure 13:**
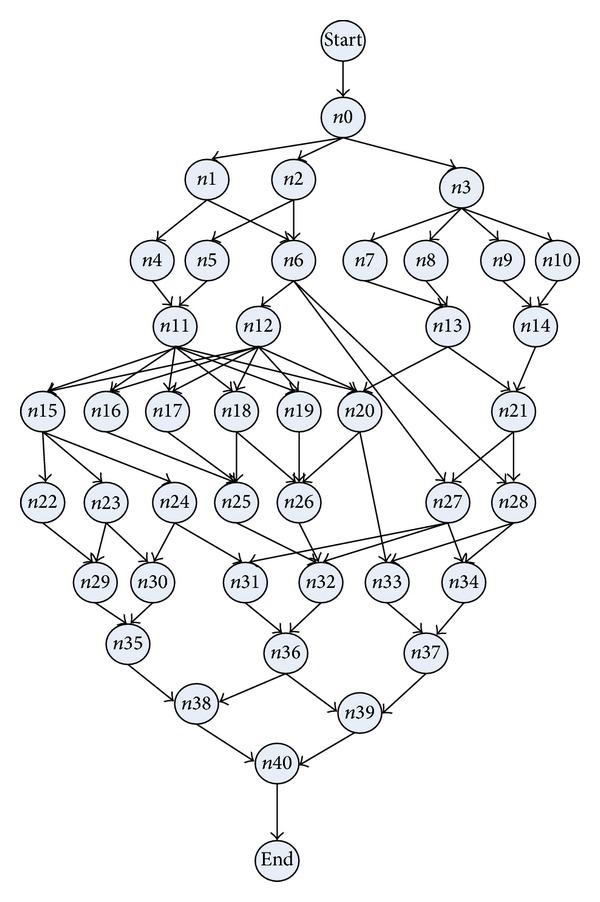
A molecular dynamics code.

**Figure 14:**
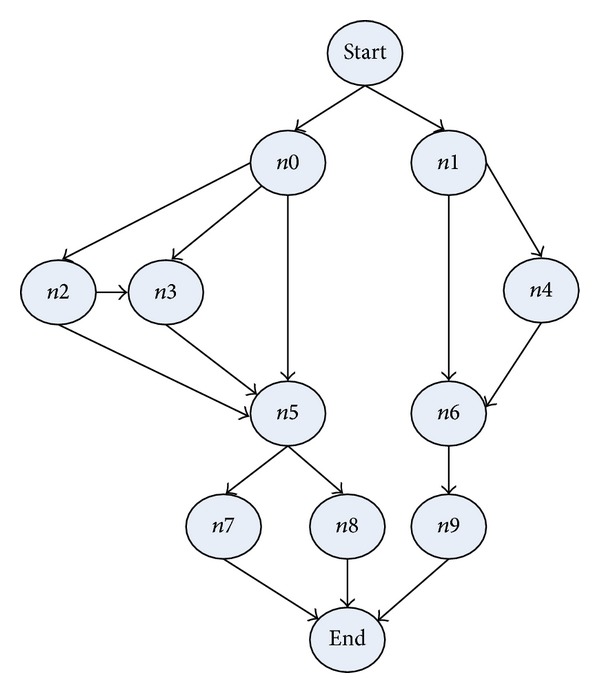
A random graph with 10 nodes.

The simulations have been performed to test TMSCRO scheduling algorithm in comparison with heuristic (HEFT_B and HEFT_T) [8] for DAG scheduling and with two metaheuristic algorithms, double molecular structure-based chemical reaction optimization (DMSCRO) [37], by using two sets of graph topology such as the real world application (Gaussian elimination and molecular dynamics code) and randomly generated application. The task graph for Gaussian elimination for input matrix of size 7 is shown in Figure 12, whereas a molecular dynamics code graph is shown in Figure 13.  Figure 14 shows a random graph with 10 nodes. The baseline performance is the makespan obtained by DMSCRO.

Considering that HEFT_B and HEFT_T have better performance than other heuristics algorithms for DAG scheduling on heterogeneous computing systems, as proposed in the 8th paragraph in Section 2.1, these two algorithms are used to be the representatives of heuristics in the simulation. There are three reasons why we regard the makespan performance of DMSCRO [37] scheduling as the baseline performance. (1) So far as we know, DMSCRO is the only one CRO-based algorithm for DAG scheduling which takes into account the searching of the task order and processor assignment. (2) As discussed in the 3rd paragraph of Section 2.2, DMSCRO [37] has the closest system model and workload to that of TMSCRO. (3) In [37], CRO-based scheduling algorithm is considered as absorbing the strengths of SA and GA. However, the underlying principles and philosophies of SA are very different from DMSCRO, and because the DMSCRO is also proved to be more effective than genetic algorithm (GA) [15] as presented in [37], we just use DMSCRO to represent the metaheuristic algorithms. We propose to make a comparison between TMSCRO and DMSCRO to validate the advantages of TMSCRO over DMSCRO.

The performance has been evaluated by the parameter makespan. The makespan values plotted in the bar graph of makespan and the chart of converge trace are, respectively, the average result of 50 and 25 independent runs to validate the robustness of TMSCRO. The communication cost is calculated by using computation costs and the computation cost ratio (CCR) values. The computation can be formulated as in (17):

(19) $$\begin{array}{c} \text{Communication}\;\text{Cost}=\text{CCR}*\text{Computation}\;\text{Cost}. \end{array}$$

All the suggested values for the other parameters of the simulation of TMSCRO and their values are listed in Table 3. These values are proposed in [20].

### 6.1. Real World Application Graphs

The real world application set is used to evaluate the performance of TMSCRO, which consists of two real world problem graph topologies, Gaussian elimination [22] and molecular dynamics code [19].

#### 6.1.1. Gaussian Elimination

Gaussian elimination is a well-known method to solve a system of linear equations. Gaussian elimination converts a set of linear equations to the upper triangular form by applying elementary row operators on them systematically. As shown in Figure 12, the matrix size of the task graph of Gaussian elimination algorithm is 7, with 27 tasks in total. In [37], this DAG has been used for the simulation of DMSCRO, and we also apply it to the evaluation of TMSCRO in this paper. Under the consideration that graph structure is fixed, the variable parameters are only 22 the communication to computation ratio (CCR) value and the heterogeneous processor number. In the simulation, CCR values were set as 0.1, 0.2, 1, 2, and 5, respectively. Considering the identical operator is executed on each processor and the information communicated between heterogeneous processors is the same in Gaussian elimination, the execution cost of each task is supposed to be the same and all communication links have the same communication cost.

The parameters and their values of the Gaussian elimination graphs performed in the simulation are given in Table 4.

**Figure 15:**
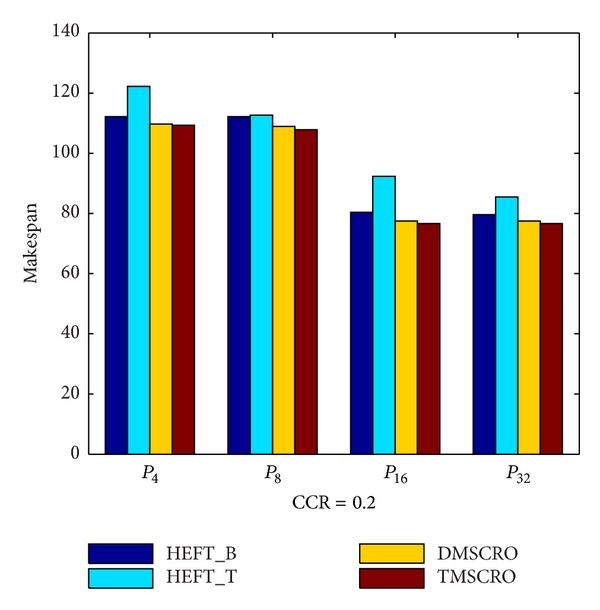
Average makespan for Gaussian elimination.

The makespan of TMSCRO, DMSCRO, HEFT_B, and HEFT_T under the increasing processor number is shown in Figure 15. As shown in Figure 15, it can also been seen that as the processor number increases, the average makespan declines, and the advantage of TMSCRO and DMSCRO over HEFT_B and HEFT_T also decreases, because when more computing nodes are contributed to run the same scale of tasks, less intelligent scheduling algorithms are needed in order to achieve good performance.

As the intelligent random search algorithms, TMSCRO and DMSCRO search a wider area of the solution space than HEFT_B, HEFT_T, or other heuristic algorithms, which narrow the search down to a very small portion of the solution space. This is the reason why TMSCRO and DMSCRO are more likely to obtain better solutions and outperform HEFT_B and HEFT_T.

The simulation results show that the performance of TMSCRO and DMSCRO is very similar to the fundamental reason that these algorithms are metaheuristic algorithms. Based on No-Free-Lunch Theorem in the field of metaheuristics, the performances of all well-designed metaheuristic search algorithms for optimal solution are the same, when averaged over all possible objective functions. The optimal solution will be gradually approached by a well-designed metaheuristic algorithm in theory, if it runs for long enough. The DMSCRO developed in [37] is well-designed, and we use it in the simulations of this paper. Therefore similar simulation results of the performances of TMSCRO and DMSCRO indicate that TMSCRO we developed is also well-designed. The detailed experiment result is shown in Table 5.

In Figure 15, the figure shows that TMSCRO is superior to DMSCRO slightly. There will be only one reason for it: the stopping criteria set in this simulation are that the makespan stays unchanged for 5000 consecutive iterations in the search loop. As discussed in the last paragraph of Section 5, all metaheuristic methods that search for optimal solutions are the same in performance when averaged over all possible objective functions. And these experimental stopping criteria make TMSCRO and DMSCRO run for long enough to gradually approach the optimal solution. Moreover, better convergence of TMSCRO makes it more efficient in searching good solutions than DMSCRO by running much less iteration times. More detailed experiment results in this regard will be presented in Section 6.3.

**Figure 16:**
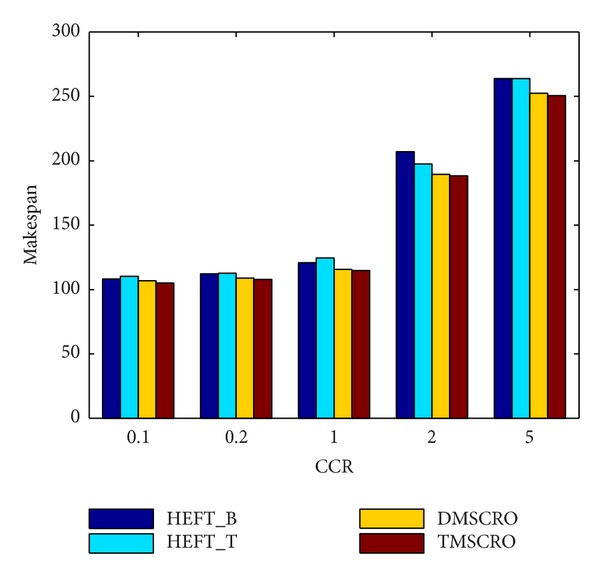
Average makespan for Gaussian elimination; the number of processors is 8.

Figure 16 shows that the average makespan of these four algorithms increases rapidly under the CCR increasing. The reason for it is because as CCR increases, the application becomes more communication intensive, making the heterogeneous processors in the idle state for longer. As shown in Figure 16, TMSCRO and DMSCRO outperform HEFT_B and HEFT_T with the advantage being more obvious as CCR becomes larger. These experimental results suggest that, for communication-intensive applications, TMSCRO and DMSCRO can deliver more consistent performance and perform more effectively than heuristic algorithms, HEFT_B and HEFT_T, in a wide range of scenarios for DAG scheduling. The detailed experiment result is shown in Table 6.

#### 6.1.2. Molecular Dynamics Code

Figure 13 shows the DAG of a molecular dynamics code as presented in [19]. As the experiment of Gaussian elimination, the structure of graph and the number of processors are fixed. The varied parameters are the number of heterogeneous processors and the CCR values which are used in our simulation are 0.1, 0.2, 1, 2, and 5.

The parameters and their values of the molecular dynamics code graphs performed in the simulation are given in Table 7.

**Figure 17:**
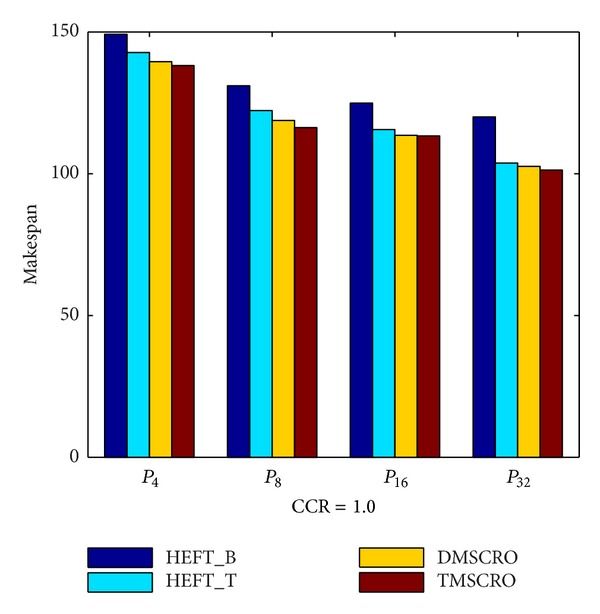
Average makespan for the molecular dynamics code.

**Figure 18:**
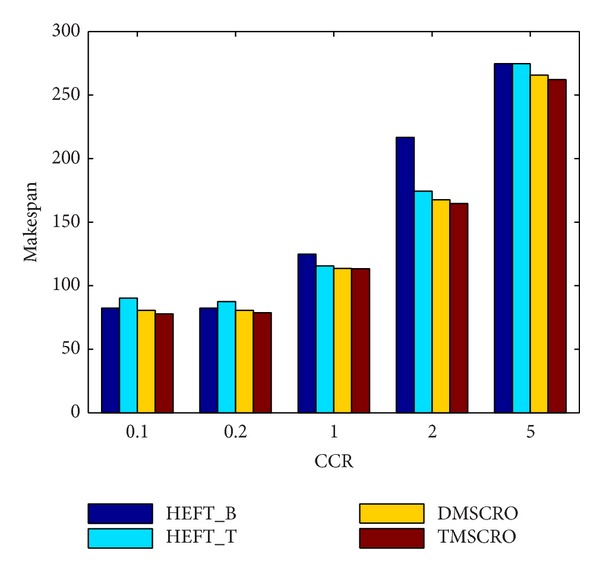
Average makespan for the molecular dynamics code; the number of processors is 16.

**Figure 19:**
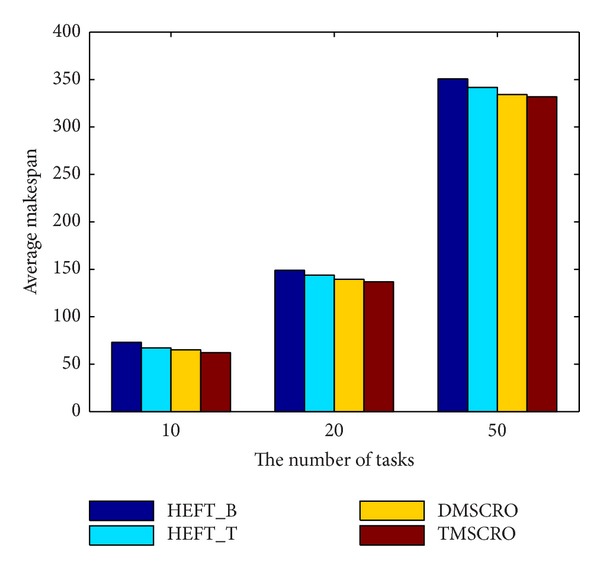
Average makespan of different task numbers, CCR = 10; the number of processors is 32.

As shown in Figures 18 and 19, under different heterogeneous processor number and different CCR values, the average makespans of TMSCRO and DMSCRO are over HEFT_B and HEFT_T, respectively. In Figure 17, it can be observed that, with the number of heterogeneous processors increasing, the average makespan decreases. The average makespan with respect to different CCR values is shown in Figure 18. The average makespan increases with the value of CCR increasing. The detailed experiment results are shown in Tables 8 and 9, respectively.

### 6.2. Random Generated Application Graphs

An effective mechanism to generate random graph for various applications is proposed in [42]. By using the probability for an edge between any two nodes, it can generate a random graph without incline towards a specific topology.

In the random graph generation of this mechanism, the topological order is used to guarantee the precedence constraints; that is, an edge exists between two nodes *v*_1_ and *v*_2_ only if *v*_1_ < *v*_2_. For probability pb, ⌊|*V*|∗pb⌋ edges are created from every node *m* to another node (*N*_1_ + (1/pb)∗*i*)mod⁡|*V*|, where 1 ≤ *i* ≤ ⌊|*V*|∗pb⌋, and ⌊*V*⌋ is the total account of task nodes in DAG.

The parameters and their values of the random graphs performed in the simulation are given in Table 10.

**Figure 20:**
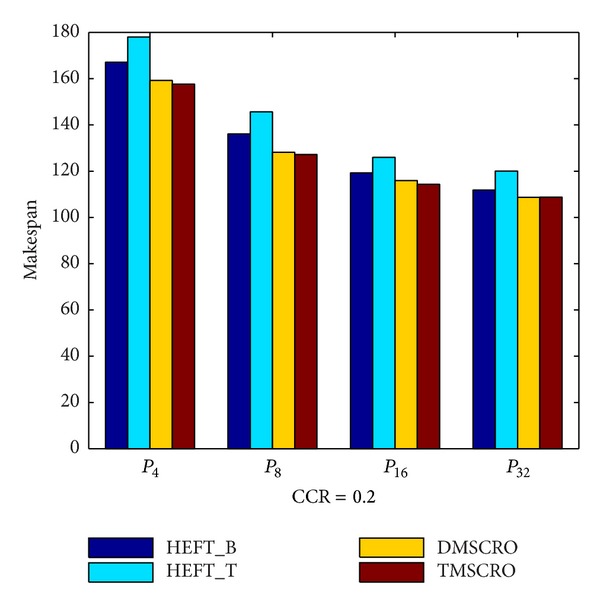
Average makespan of four algorithms under different processor numbers and the low communication costs; the number of tasks is 50.

**Figure 21:**
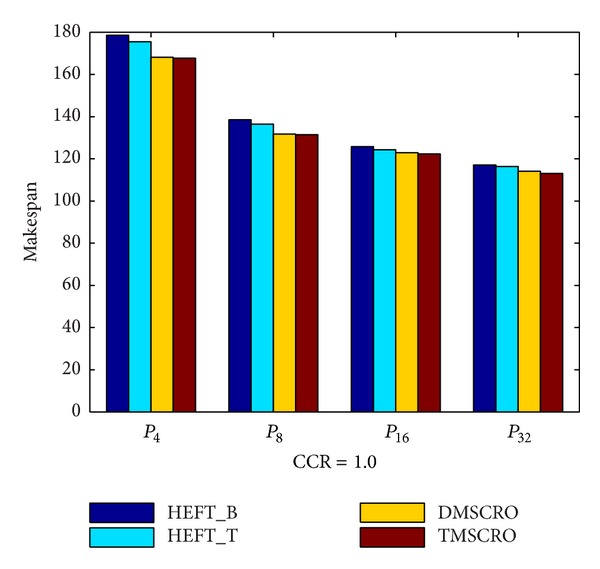
Average makespan of four algorithms under different processor numbers and the low communication costs; the number of tasks is 50.

Figure 19 shows that TMSCRO always outperforms HEFT_B, HEFT_T, and DMSCRO with the number of tasks in a DAG increasing. The comparison of the average makespan of four algorithms under the increase of heterogeneous processor number is shown in Figures 20 and 21. As can be seen from these figures, the performance of TMSCRO is better than the other three algorithms in all cases. The reasons for these two figures are the same as those explained in Figure 15. The detailed experiment results are shown in Tables 11, 12, and 13, respectively.

**Figure 22:**
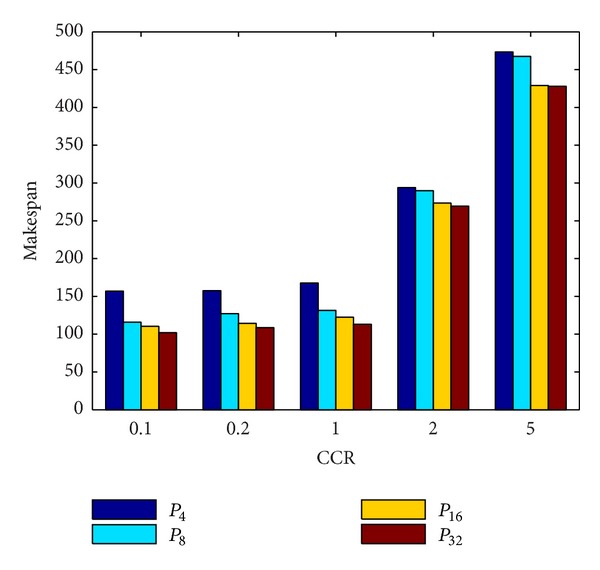
Average makespan of TMSCRO under different values of CCR; the number of tasks is 50.

As shown in Figure 22, it can be observed that the average makespan approached by TMSCRO increases rapidly with CCR values increasing. This may be because as CCR increases, the application becomes more communication intensive, making the heterogeneous processors in the idle state for longer. The detailed experiment results are shown in Table 14.

### 6.3. Convergence Trace of TMSCRO

The result of the experiments in the previous subsections is the final makespan obtained by TMSCRO and DMSCRO, showing that TMSCRO can obtain similar makespan performance as DMSCRO. Moreover, in some cases the final makespan achieved by TMSCRO is even better than that by DMSCRO after the stop criteria are satisfied. In this section, the change of makespan in the experiments as TMSCRO and DMSCRO progress during the search is demonstrated by comparing the convergence trace of these two algorithms. These experiments help further reveal the better performance of TMSCRO on convergence and can also help explain why the TMSCRO sometimes outperforms DMSCRO in some cases.

The parameters and their values of the Gaussian elimination, molecular dynamics code, and random graphs performed in the simulation are given in Tables 15, 16, and 17, respectively.

**Figure 23:**
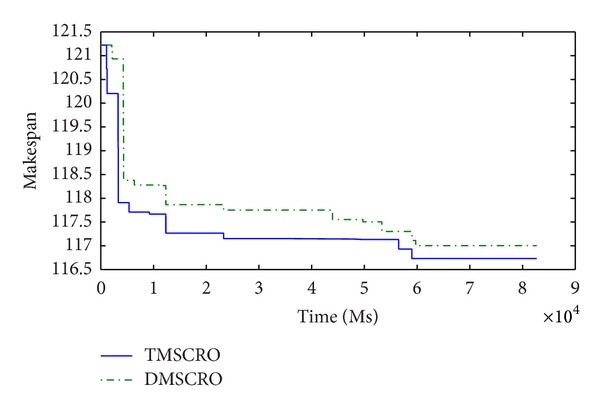
The convergence trace for Gaussian elimination; ccr = 0.2; the number of processors is 8.

**Figure 24:**
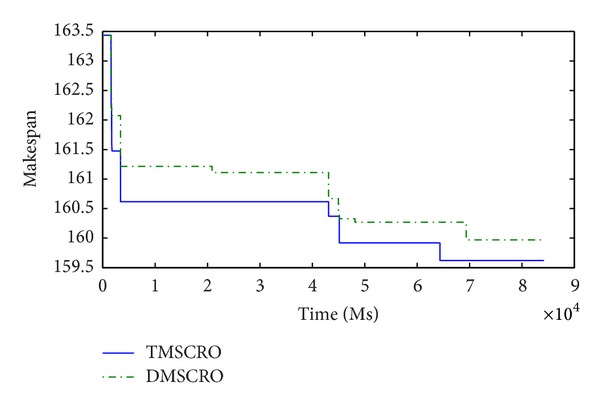
The convergence trace for the molecular dynamics code; ccr = 1; the number of processors is 16.

**Figure 25:**
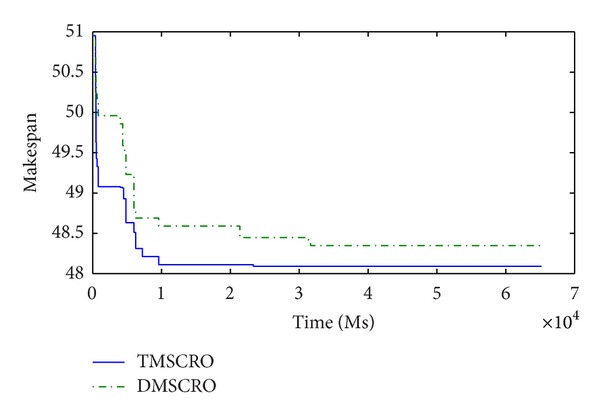
The convergence trace for the randomly generated DAGs with each containing 10 tasks.

**Figure 26:**
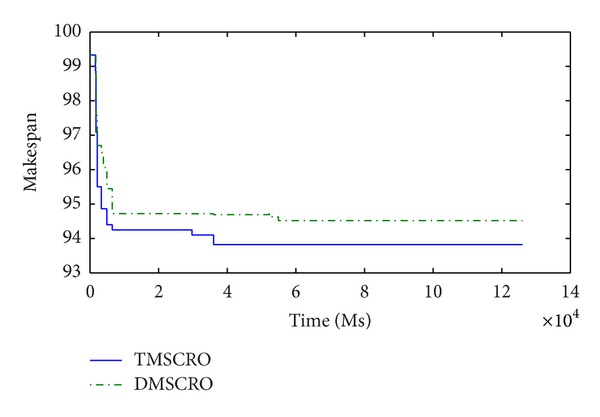
The convergence trace for the randomly generated DAGs with each containing 20 tasks.

**Figure 27:**
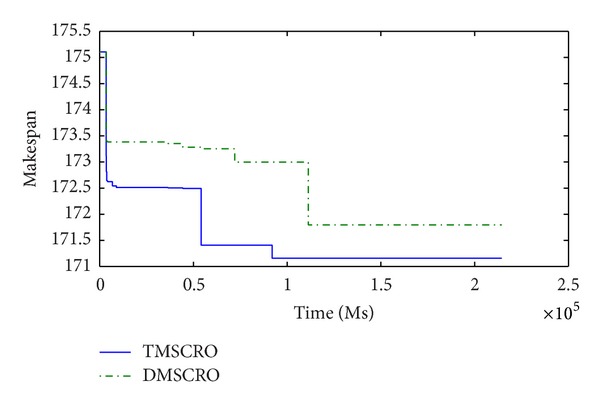
The convergence trace for the randomly generated DAGs with each containing 50 tasks.

Figures 23 and 24, respectively, plot the convergence traces for processing Gaussian elimination and the molecular dynamics code. Figures 25, 26, and 27  show the convergence traces when processing the sets of randomly generated DAGs and each set contains the DAGs of 10, 20, and 50 tasks, respectively. These figures demonstrated that the makespan performance decreases quickly as both TMSCRO and DMSCRO progress and that the decreasing trends tail off when the algorithms run for long enough. These figures also show that, in most cases, the convergence traces of both algorithms are rather different even though the final makespans obtained by them are almost the same.

The statistical analysis results over the average coverage rate at 5000 ascending sampling points from start time to end time of all the experiments are shown in Table 18 (the threshold of *P* is set as 0.05), which are obtained by Friedman test, and each experiment is carried out 25 times. We can find that the differences between two algorithms in performance are significant from a statistical point of view. The reason of it is because the super molecule makes TMSOCRO have a stronger convergence capability, especially early in each run. Moreover, the performance of TMSCRO on convergence is better than DMSCRO. Quantitatively, our records show that TMSCRO converges faster than DMSCRO by 12.89% on average in all the cases (by 23.27% on average in the best case).

In these experiments, the stopping criteria of the algorithms are that the algorithm stops when the makespan performance remains unchanged for a preset number of consecutive iterations in the search loop (in the experiments, it is 5000 iterations). In reality, the algorithms can also stop when the total processing time of it reaches a preset value (e.g., 180s). Moreover, both of TMSCRO and DMSCRO have the same initial population. In this case, the fact that TMSCRO outperforms DMSCRO on convergence means that the makespan achieved by TMSCRO could be much better than that by DMSCRO when the stopping criteria of the algorithm are satisfied. The reason for this can be explained by the analysis presented in the last paragraph of Section 5.3.

## 7. Conclusion

In this paper, we developed a TMSCRO for DAG scheduling on heterogeneous systems based on chemical reaction optimization (CRO) method. With a more reasonable reaction molecular structure and four designed elementary chemical reaction operators, TMSCRO has a better ability on intensification and diversification search than DMSCRO, which is the only one CRO-based algorithm for DAG scheduling on heterogeneous systems as far as we know. Moreover, in TMSCRO, the algorithm constrained earliest finish time (CEFT) and constrained-critical-path directed acyclic graph (CCPDAG) are applied to the data pretreatment, and the concept of constrained paths (CCPs) is also utilized in the initialization. We also use the first initial molecule, InitS, to be a super molecule for accelerating convergence. As a metaheuristic method, the TMSCRO algorithm can cover a much larger search space than heuristic scheduling approaches. The experiments show that TMSCRO outperforms HEFT_B and HEFT_T and can achieve a higher speedup of task executions than DMSCRO.

In future work, we plan to extend TMSCRO by applying synchronous communication strategy to parallelize the processing of TMSCRO. This kind of design will divide the molecules into groups and each group of molecules is handled by a CPU or GPU. So, multiple groups can be manipulated simultaneously in parallel and molecules can also be exchanged among the CPUs or GPUs from time to time in order to reduce the time cost.

## Figures and Tables

**Algorithm 1:**
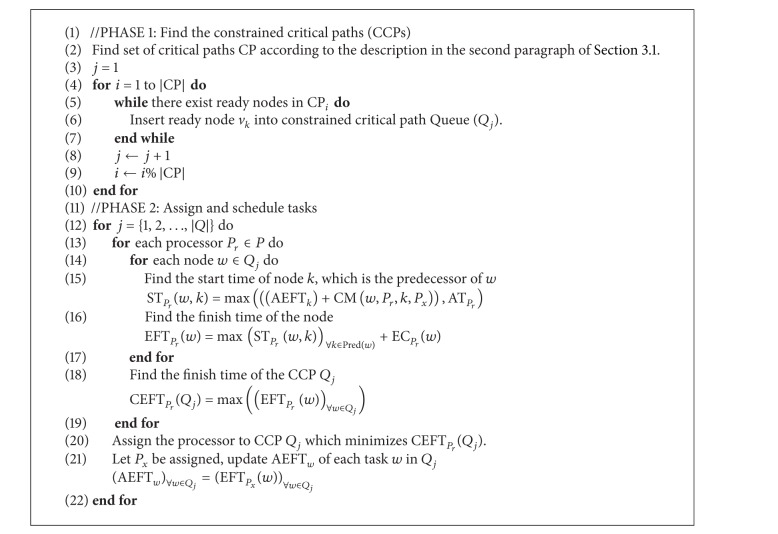
CEFT.

**Algorithm 2:**
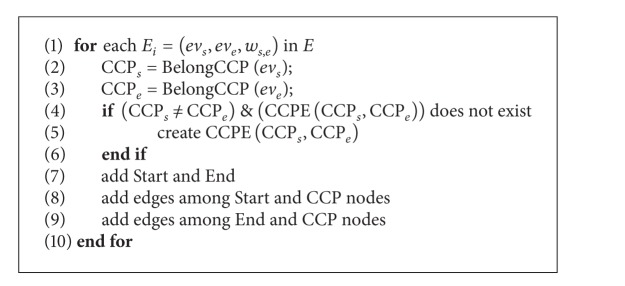
Gen_CCPDAG(DAG, CCP) generating CCPDAG.

**Algorithm 3:**
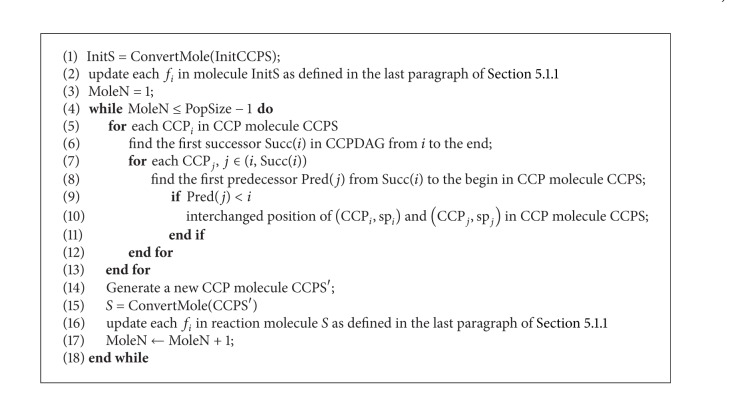
InitTMolecule(InitCCPS) generating the initial population.

**Algorithm 4:**
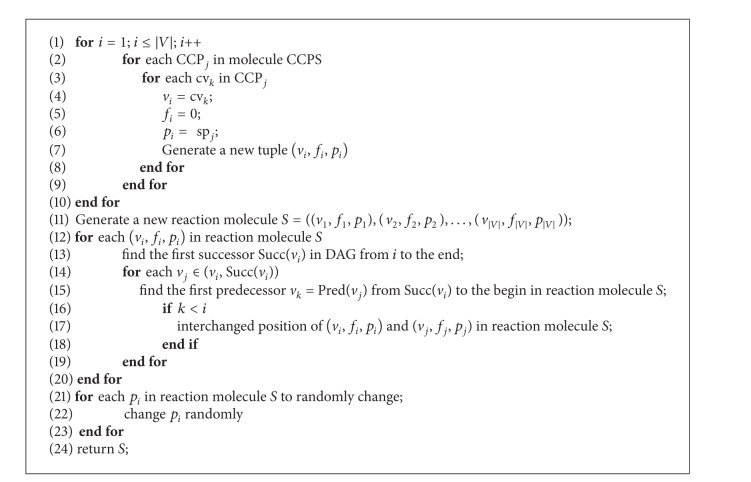
ConvertMole(CCPS) converting a CCPS to an *S*.

**Algorithm 5:**
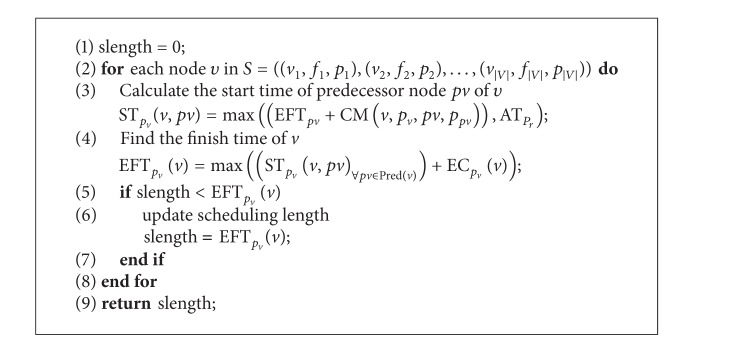
Fit(S) calculating the fitness value of a molecule and the processor allocation optimization.

**Algorithm 6:**
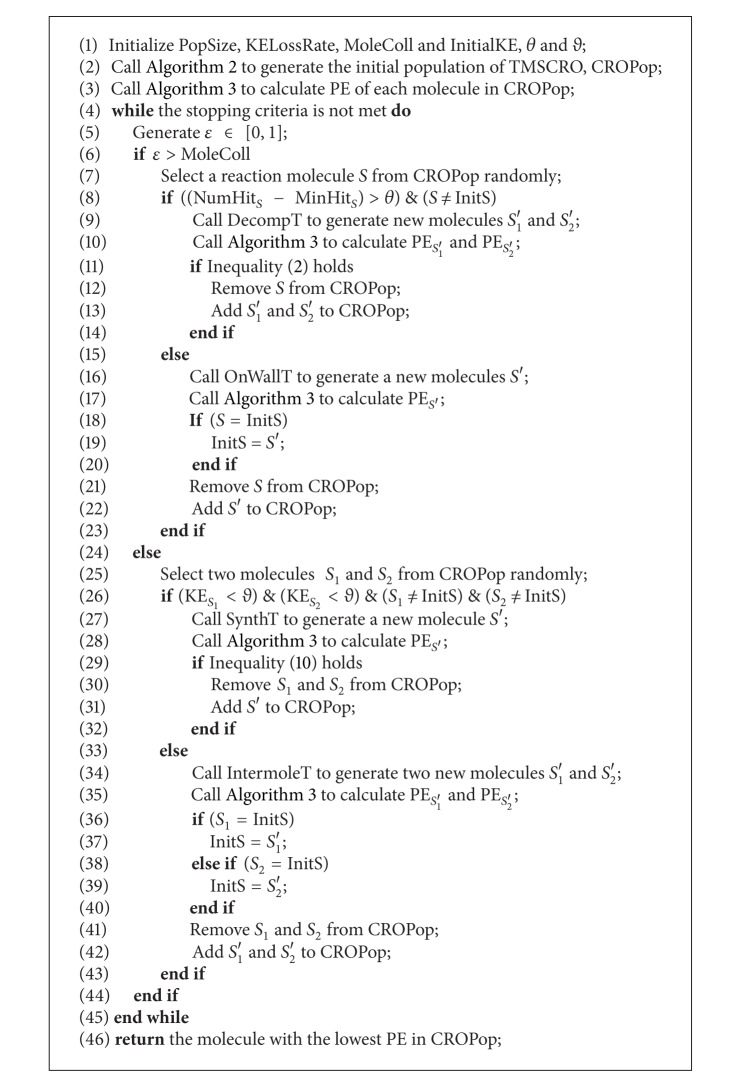
TMSCRO(DAG) The TMSCRO outline(framework).

**Table 1:** Specific terms and their usage for the CEFT algorithm.

EC_*P*_*r*__(*w*)	Execution cost of a node *w* using processor *P*_*r*_
CM(*w*, *P*_*r*_, *v*, *P*_*x*_)	Communication cost from node *v* to *w*, if *P*_*x*_ has been assigned to node *v* and *P*_*r*_ is assigned to node *w*
ST_*P*_*r*__(*w*, *v*)	Possible start time of node *w* which is assigned the processor *P*_*r*_ with the *v* node being any predecessor of *w* which has already been scheduled
EFT_*P*_*r*__(*w*)	Finish time of node *w* using processor *P*_*r*_
AEFT_*w*_	Actual finish time of node *w*
CEFT_*P*_*r*__(CCP_*j*_)	Finish time of the constrained critical path *Q*_*j*_ when processor *P*_*r*_ is assigned to it
AT_*P*_*r*__	Availability time of *P*_*r*_
Pred(*w*)	Set of predecessors of node *w*
Succ(*w*)	Set of successors of node *w*
AEC(*w*)	Average execution cost of node *w*

**Table 2:** CCP corresponding to the DAG as shown in Figure 1(1).

*i*	CCP_*i*_
1	A-B-D
2	C-G
3	F
4	E
5	H
6	I
7	J

**Table 3:** Configuration parameters for the simulation of TMSCRO.

Parameter	Value
InitialKE	1000
*θ*	500
*ϑ*	10
Buffer	200
KELossRate	0.2
MoleColl	0.2
PopSize	10
*g*	0.33
Number of runs	50

**Table 4:** Configuration parameters for the Gaussian elimination graphs.

Parameter	Possible values
CCR	{0.1, 0.2, 1, 2, 5}
Number of processors	{4, 8, 16, 32}
Number of tasks	27

**Table 5:** The experiment results for the Gaussian elimination graph under different processors, CCR = 0.2.

The number of processors	HEFT_B (the average makespan)	HEFT_T (the average makespan)	DMSCRO (the average makespan)	TMSCRO (the average makespan)	TMSCRO (the best makespan)	TMSCRO (the worst makespan)	TMSCRO (the variance of resultant makespans)
4	112.2	122.227	109.9	109.31	109.2	109.9	0.2473
8	112.2	112.648	108.9	107.83	107.1	108.9	0.9613
16	80.4	92.354	77.5	76.62	76.3	78.9	1.6696
32	79.64	85.454	77.5	76.62	76.1	78.9	1.7201

**Table 6:** The experiment results for the Gaussian elimination graph under different CCRs; the number of processors is 8.

CCR	HEFT_B (the average makespan)	HEFT_T (the average makespan)	DMSCRO (the average makespan)	TMSCRO (the average makespan)	TMSCRO (the best makespan)	TMSCRO (the worst makespan)	TMSCRO (the variance of resultant makespans)
0.1	108.2	110.312	106.78	105.04	104.76	106.6	1.7271
0.2	112.2	112.648	108.9	107.83	107.1	108.9	0.9613
1	120.752	124.536	115.63	114.717	114.3	115.4	0.3787
2	207.055	197.504	189.4	188.303	188.1	188.75	0.1522
5	263.8	263.8	252.39	250.671	250.3	251.79	0.9178

**Table 7:** Configuration parameters for the molecular dynamics code graphs.

Parameter	Possible values
CCR	{0.1, 0.2, 1, 2, 5}
Number of processors	{4, 8, 16, 32}
Number of tasks	41

**Table 8:** The experiment results for the molecular dynamics code graph under different processors, CCR = 1.0.

The number of processors	HEFT_B (the average makespan)	HEFT_T (the average makespan)	DMSCRO (the average makespan)	TMSCRO (the average makespan)	TMSCRO (the best makespan)	TMSCRO (the worst makespan)	TMSCRO (the variance of resultant makespans)
4	149.205	142.763	139.51	138.13	137.87	138.6	0.1749
8	131.031	122.265	118.8	116.9	116.2	117.33	0.2764
16	124.868	115.584	113.52	113.36	113.1	113.43	0.0237
32	120.047	103.784	102.617	101.29	101.023	101.47	0.0442

**Table 9:** The experiment results for the molecular dynamics code graph under different CCRs; the number of processors is 16.

CCR	HEFT_B (the average makespan)	HEFT_T (the average makespan)	DMSCRO (the average makespan)	TMSCRO (the average makespan)	TMSCRO (the best makespan)	TMSCRO (the worst makespan)	TMSCRO (the variance of resultant makespans)
0.1	82.336	90.136	80.53	77.781	77.3	78.9	0.9459
0.2	82.356	87.504	80.53	78.704	78.21	79.13	0.2002
1	124.868	115.584	113.52	113.36	113.1	113.43	0.0237
2	216.735	174.501	167.612	164.7	164.32	164.91	0.0742
5	274.7	274.7	265.8	262.173	262.022	262.6	0.1344

**Table 10:** Configuration parameters for random graphs.

Parameter	Possible values
CCR	{0.1, 0.2, 1, 2, 5, 10}
Number of processors	{4, 8, 16, 32}
Number of tasks	{10, 20, 50}

**Table 11:** The experiment results for the random graph under different task numbers, CCR = 10; the number of processors is 32.

The number of tasks	TMSCRO (the average makespan)	TMSCRO (the best makespan)	TMSCRO (the worst makespan)	TMSCRO (the variance of resultant makespans)
10	73	67	65.1	62.2
20	148.9	143.9	139.421	136.8
50	350.7	341.7	334.17	331.9

**Table 12:** The experiment results for the random graph under different processors, CCR = 0.2; the number of tasks is 50.

The number of processors	HEFT_B (the average makespan)	HEFT_T (the average makespan)	DMSCRO (the average makespan)	TMSCRO (the average makespan)	TMSCRO (the best makespan)	TMSCRO (the worst makespan)	TMSCRO (the variance of resultant makespans)
4	167.12	178.023	159.234	157.63	157.12	158.3	0.3923
8	136.088	145.649	128.17	127.178	127.06	127.7	0.1949
16	119.292	125.986	115.9	114.33	114.1	115.2	0.4753
32	111.866	120.065	108.7	108.71	108.31	108.9	0.0733

**Table 13:** The experiment results for the random graph under different processors, CCR = 1.0; the number of tasks is 50.

The number of processors	HEFT_B (the average makespan)	HEFT_T (the average makespan)	DMSCRO (the average makespan)	TMSCRO (the average makespan)	TMSCRO (the best makespan)	TMSCRO (the worst makespan)	TMSCRO (the variance of resultant makespans)
4	178.662	175.52	168.12	167.703	167.42	168	0.0857
8	138.572	136.47	131.8	131.451	131.1	131.9	0.178
16	125.772	124.31	122.91	122.32	122.1	122.432	0.0233
32	117.11	116.4	114.124	113.127	112.9	113.54	0.1348

**Table 14:** The experiment results for the random graph under different task CCRs, the number of tasks is 50.

CCR	The number of processors is 4	The number of processors is 8	The number of processors is 16	The number of processors is 32
0.1	156.97	115.724	110.3	101.87
0.2	157.63	127.178	114.33	108.71
1	167.703	131.451	122.32	113.127
2	294.042	289.878	273.375	269.514
5	473.5	467.61	429.13	428.13

**Table 15:** Configuration parameters of convergence experiment for the Gaussian elimination graph.

Parameter	Value
CCR	0.2
Number of processors	8
Number of tasks	27

**Table 16:** Configuration parameters of convergence experiment for the molecular dynamics graph.

Parameter	Value
CCR	1
Number of processors	16
Number of tasks	41

**Table 17:** Configuration parameters of convergence experiment for the random graphs.

Parameter	Values
CCR	{0.2, 1}
Number of processors	{8, 16}
Number of tasks	{10, 20, 50}

**Table 18:** The results of the statistical analysis over the average coverage rate at different sampling times of all the experiments (the threshold of **P** is set as** 0.05**).

DAG	The value of *P* after Friedman test	Average convergence acceleration ratio
Gaussian elimination	7.10 × 10^−8^	4.23%
Molecular dynamics code	2.54 × 10^−8^	7.21%
Random graph with 10 tasks	4.26 × 10^−8^	23.27%
Random graph with 20 tasks	3.48 × 10^−8^	16.41%
Random graph with 50 tasks	2.58 × 10^−8^	13.32%

## Notations

DAG = (*V*, *E*): Input directed acyclic graph with |*V*| nodes representing tasks, and |*E*| edges representing constrained relations among the tasks
*V* = (*v*_1_, *v*_2_,…, *v*_|*V*|_): Node sequence in which the hypothetical entry node (with no predecessors) *v*_1_ and end node (with no successors) *v*_|*V*|_, respectively, represent the beginning and end of execution
*E* = {*E*_*i*_∣*i* = 1,2, 3,…, |*E*|}: Edge set in which *E*_*i*_ = (ev_*s*_, ev_*e*_, ew_*s*,*e*_), with ev_*s*_ & ev_*e*_ ∈ {*v*_1_, *v*_2_,…, *v*_|*V*|_} representing its start and end nodes, and the value of communication cost between ev_*s*_ and ev_*e*_ denoted as ew_*s*,*e*_
*P* = {*p*_*i*_∣*i* = 1,2, 3,…, |*P*|}: Set of multiple heterogeneous processors in target system
CCP = (CCP_1_, CCP_2_,…, CCP_|CCP|_): Constrained-critical-path sequence of DAG = (*V*, *E*)
CCP_*i*_ = (cv_*i*,1_, cv_*i*,2_,…, cv_*i*,|CCP_*i*_|_): Constrained critical path in which the set {cv_*i*,1_, cv_*i*,2_,…, cv_*i*,|CCP_*i*_|_}⊆{*v*_1_, *v*_2_,…, *v*_|*V*|_}
CCPDAG: Directed acyclic graph with |CCP| nodes representing CCPs, two virtual nodes (i.e., start and end) representing the beginning and exit of execution, respectively, and |CE| edges representing dependencies among all nodes
CCPS = ((CCP_1_, sp_1_), (CCP_2_, sp_2_),…, (CCP_|CCP|_, sp_|CCP|_)): A CCP molecule used in the initialization of TMSCRO, in which sp_*i*_ is the processor assigned to the constrained-critical-path CCP_*i*_
*S* = ((*v*_1_, *f*_1_, *p*_1_), (*v*_2_, *f*_2_, *p*_2_),…,(*v*_|*V*|_, *f*_|*V*|_, *p*_|*V*|_)): A reaction molecule (i.e., solution) in TMSCRO
(*v*_*i*_, *f*_*i*_, *p*_*i*_): Atom (i.e., tuple) in *S*
InitCCPS: The first CCP molecule for the initialization of TMSCRO
InitS: The first molecule in TMSCRO
BelongCCP(*w*): CCP_*i*_ that node *w* belongs to
CCPE(CCPs, CCP_*e*_): Edge between CCPs and CCPe
$\overset{-}{W(v)}$: Average computation cost of node *v*
EC_*P*_*r*__(*w*): Execution cost of a node *w* using processor *P*_*r*_
CM(*w*, *P*_*r*_, *v*, *P*_*x*_): Communication cost from node *v* to *w*, if *P*_*x*_ has been assigned to node *v* and *P*_*r*_ is assigned to node *w*
ST_*P*_*r*__(*w*, *v*): Possible start time of node *w* which is assigned the processor *P*_*r*_ with the *v* node being any predecessor of *w* which has already been scheduled
EFT_*P*_*r*__(*w*): Finish time of node *w* using processor *P*_*r*_
AT_*P*_*r*__: Availability time of *P*_*r*_
Pred(*w*): Set of predecessors of node *w*
Succ(*w*): Set of successors of node *w*
CCR: Communication to computation ratio
*g*: The parameter to adjust the heterogeneity level in a heterogeneous system
PE: Current potential energy of a molecule
KE: Current kinetic energy of a molecule
InitialKE: Initial kinetic energy of a molecule
*θ*: Threshold value guiding the choice of on-wall collision or decomposition
*ϑ*: Threshold value guiding the choice of intermolecule collision or synthesis
Buffer: Initial energy in the central energy buffer
KELossRate: Loss rate of kinetic energy
MoleColl: Threshold value to determine whether to perform a unimolecule reaction or an intermolecule reaction
PopSize: Size of the molecules
NumHit: Total collision number of a molecule.

## References

[1] Graham JLRL, Lawler EL, Kan AR (1979). Optimization and approximation in deterministic sequencing and scheduling: a survey. *Annals of Discrete Mathematics*.

[2] Papadimitriou C, Yannakakis M Towards an architecture-independent analysis of parallel algorithms.

[3] Sarkar V (1989). *Partitioning and Scheduling Parallel Programs for Multiprocessors*.

[4] Chrétienne P (1992). Task scheduling with interprocessor communication delays. *European Journal of Operational Research*.

[5] Khan MA (2012). Scheduling for heterogeneous systems using constrained critical paths. *Parallel Computing*.

[6] Xu J, Albert Lam YS, Victor Li OK Stock portfolio selection using chemical reaction optimization.

[7] Kwok Y-K, Ahmad I (1999). Static scheduling algorithms for allocating directed task graphs to multiprocessors. *ACM Computing Surveys*.

[8] Topcuoglu H, Hariri S, Wu M-Y (2002). Performance-effective and low-complexity task scheduling for heterogeneous computing. *IEEE Transactions on Parallel and Distributed Systems*.

[9] Amini A, Wah TY, Saybani MR, Yazdi SRAS A study of density-grid based clustering algorithms on data streams.

[10] Cheng H A high efficient task scheduling algorithm based on heterogeneous multi-core processor.

[11] Tsuchiya T, Osada T, Kikuno T A new heuristic algorithm based on gas for multiprocessor scheduling with task duplication.

[12] Bajaj R, Agrawal DP (2004). Improving scheduling of tasks in a heterogeneous environment. *IEEE Transactions on Parallel and Distributed Systems*.

[13] Ge H-W, Sun L, Liang Y-C, Qian F (2008). An effective PSO and AIS-based hybrid intelligent algorithm for job-shop scheduling. *IEEE Transactions on Systems, Man, and Cybernetics A: Systems and Humans*.

[14] Ho NB, Tay JC (2008). Solving multiple-objective flexible job shop problems by evolution and local search. *IEEE Transactions on Systems, Man and Cybernetics C: Applications and Reviews*.

[15] Hou ESH, Ansari N, Ren H (1994). Genetic algorithm for multiprocessor scheduling. *IEEE Transactions on Parallel and Distributed Systems*.

[16] Hwang J-J, Chow Y-C, Anger FD, Lee C-Y (1989). Scheduling precedence graphs in systems with interprocessor communication times. *SIAM Journal on Computing*.

[17] Iverson M, Özgüner F, Follen G Parallelizing existing applications in a distributed heterogeneous environment.

[18] Kashani MH, Jahanshahi M Using simulated annealing for task scheduling in distributed systems.

[19] Kim S, Browne J A general approach to mapping of parallel computation upon multiprocessor architectures.

[20] Lam AYS, Li VOK (2010). Chemical-reaction-inspired metaheuristic for optimization. *IEEE Transactions on Evolutionary Computation*.

[21] Li H, Wang L, Liu J Task scheduling of computational grid based on particle swarm algorithm.

[22] Wu M-Y, Gajski DD (1990). Hypertool: a programming aid for message-passing systems. *IEEE Transactions on Parallel and Distributed Systems*.

[23] Sih GC, Lee EA (1993). Compile-time scheduling heuristic for interconnection-constrained heterogeneous processor architectures. *IEEE Transactions on Parallel and Distributed Systems*.

[24] El-Rewini H, Lewis TG (1990). Scheduling parallel program tasks onto arbitrary target machines. *Journal of Parallel and Distributed Computing*.

[25] Lin F-T (2002). Fuzzy job-shop scheduling based on ranking level (lambda, 1) interval-valued fuzzy numbers. *IEEE Transactions on Fuzzy Systems*.

[26] Liu B, Wang L, Jin Y-H (2007). An effective PSO-based memetic algorithm for flow shop scheduling. *IEEE Transactions on Systems, Man, and Cybernetics B: Cybernetics*.

[27] Pop F, Dobre C, Cristea V Genetic algorithm for DAG scheduling in Grid environments.

[28] Shanmugapriya R, Padmavathi S, Shalinie SM Contention awareness in task scheduling using tabu search.

[29] Shi L, Pan Y (2005). An efficient search method for job-shop scheduling problems. *IEEE Transactions on Automation Science and Engineering*.

[30] Choudhury P, Kumar R, Chakrabarti PP (2008). Hybrid scheduling of dynamic task graphs with selective duplication for multiprocessors under memory and time constraints. *IEEE Transactions on Parallel and Distributed Systems*.

[31] Song S, Hwang K, Kwok Y-K (2006). Risk-resilient heuristics and genetic algorithms for security-assured grid job scheduling. *IEEE Transactions on Computers*.

[32] Spooner DP, Cao J, Jarvis SA, He L, Nudd GR (2005). Performance-aware workflow management for grid computing. *The Computer Journal*.

[33] Li K, Tang X, Li K (2014). Energy-efficient stochastic task scheduling on heterogeneous computing systems. *IEEE Transactions on Parallel and Distributed Systems*.

[34] Wang J, Duan Q, Jiang Y, Zhu X A new algorithm for grid independent task schedule: genetic simulated annealing.

[35] He L, Zou D, Zhang Z, Chen C, Jin H, Jarvis S (2012). Developing resource consolidation frameworks for moldable virtual machines in clouds. *Future Generation Computer Systems*.

[36] Xu Y, Li K, Hu J, Li K (2014). A genetic algorithm for task scheduling on heterogeneous computing systems using multiple priority queues. *Information Sciences*.

[37] Xu Y, Li K, He L, Truonga TK (2013). A DAG scheduling scheme on heterogeneous computing systems using double molecular structure-based chemical reaction optimization. *Journal of Parallel and Distributed Computing*.

[38] Xu J, Lam A, Li V Chemical reaction optimization for the grid scheduling problem.

[39] Varghese B, Mckee G, Alexandrov V (2011). Can agent intelligence be used to achieve fault tolerant parallel computing systems?. *Parallel Processing Letters*.

[40] Xu J, Lam A, Li V (2011). Chemical reaction optimization for task scheduling in grid computing. *IEEE Transactions on Parallel and Distributed Systems*.

[41] Truong TK, Li K, Xu Y (2013). Chemical reaction optimization with greedy strategy for the 0-1 knapsack problem. *Applied Soft Computing Journal*.

[42] Almeida VAF, Vasconcelos IMM, Arabe JNC, Menasce DA Using random task graphs to investigate the potential benefits of heterogeneity in parallel systems.

